# Ketone body **β**-hydroxybutyrate is an autophagy-dependent vasodilator

**DOI:** 10.1172/jci.insight.149037

**Published:** 2021-10-22

**Authors:** Cameron G. McCarthy, Saroj Chakraborty, Gagandeep Singh, Beng San Yeoh, Zachary J. Schreckenberger, Avinash Singh, Blair Mell, Nicole R. Bearss, Tao Yang, Xi Cheng, Matam Vijay-Kumar, Camilla F. Wenceslau, Bina Joe

**Affiliations:** Center for Hypertension and Personalized Medicine, Department of Physiology and Pharmacology, College of Medicine and Life Sciences, University of Toledo, Toledo, Ohio, USA.

**Keywords:** Vascular Biology, Cardiovascular disease, Microcirculation, Pharmacology

## Abstract

Autophagy has long been associated with longevity, and it is well established that autophagy reverts and prevents vascular deterioration associated with aging and cardiovascular diseases. Currently, our understanding of how autophagy benefits the vasculature is centered on the premise that reduced autophagy leads to the accumulation of cellular debris, resulting in inflammation and oxidative stress, which are then reversed by reconstitution or upregulation of autophagic activity. Evolutionarily, autophagy also functions to mobilize endogenous nutrients in response to starvation. Therefore, we hypothesized that the biosynthesis of the most physiologically abundant ketone body, β-hydroxybutyrate (βHB), would be autophagy dependent and exert vasodilatory effects via its canonical receptor, Gpr109a. To the best of our knowledge, we have revealed for the first time that the biosynthesis of βHB can be impaired by preventing autophagy. Subsequently, βHB caused potent vasodilation via potassium channels but not Gpr109a. Finally, we observed that chronic consumption of a high-salt diet negatively regulates both βHB biosynthesis and hepatic autophagy and that reconstitution of βHB bioavailability prevents high-salt diet–induced endothelial dysfunction. In summary, this work offers an alternative mechanism to the antiinflammatory and antioxidative stress hypothesis of autophagy-dependent vasculoprotection. Furthermore, it reveals a direct mechanism by which ketogenic interventions (e.g., intermittent fasting) improve vascular health.

## Graphical abstract

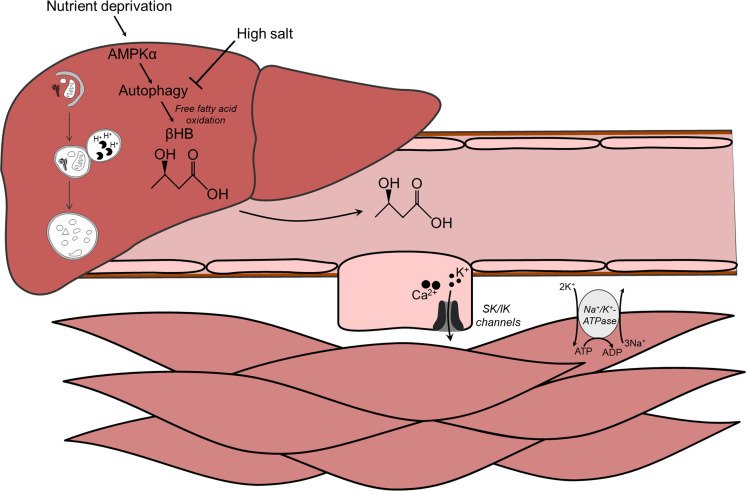

## Introduction

Autophagy is the constitutively active catabolic process essential for maintaining homeostasis via the degradation and recycling of cellular debris and dysfunctional organelles (1). While autophagy has long been associated with health and longevity for multiple compelling reasons (2), including the lengthening of life span (3), a decline in autophagy has been linked to the deterioration of organ function via the buildup of toxic cellular waste. Indeed, this notion is supported by a number of studies that have demonstrated that autophagy-promoting lifestyle interventions, pharmacological activators, and transgenic overexpression of autophagy genes all reverse phenotypes of vascular deterioration (4–6), in both aged (7, 8) and hypertensive (9) laboratory rodents.

In addition to the clearance of cellular waste, autophagy is also closely linked to metabolic homeostasis (10). It is well known that autophagy can mobilize endogenous macro- and micronutrients in times of starvation and stress (11). The liver is our largest metabolic organ and is primarily responsible for endogenously stored energy substrates (12). It has been established that hepatic autophagy mediates lipid droplet and triglyceride breakdown, and inhibition of autophagy increases lipid storage in the liver (11). Thus, autophagy can promote the liberation of free fatty acids that could subsequently be oxidized for ketone body biosynthesis (13). The most abundant circulating ketone body is β-hydroxybutyrate (βHB), an endogenous carrier of energy from the liver to peripheral tissues when exogenous nutrients wane, such as under conditions of caloric restriction or intermittent fasting. Our laboratory has recently reported that Dahl salt–sensitive (Dahl S) rats fed a high-salt diet synthesize less βHB in response to a 24-hour fast compared with Dahl S rats fed a low-salt diet and that reconstituting βHB bioavailability had profound antihypertensive effects (14). Nonetheless, the mechanistic underpinnings of these phenotypes have not been fully explored, nor has the direct effect of βHB on vascular function.

In addition to being an energy source, βHB can also serve as a signaling molecule, primarily through activation of the GPCR Gpr109a (also known as hydroxycarboxylic acid receptor 2, niacin receptor 1, or PUMA-G) and inhibition of Gpr41 (also known as free fatty acid receptor 3) (15). Therefore, the major hypothesis for the current investigation was that upregulation of autophagy in the liver after fasting stimulates the production of βHB, which induces vasodilation via endothelial Gpr109a. Furthermore, a high-salt diet will decrease autophagy and βHB biosynthesis, subsequently contributing to vascular damage. These hypotheses reveal a potentially novel physiologic mechanism by which autophagy is vasculoprotective as well as a pathophysiologic mechanism of vascular deterioration after consuming a high-salt diet.

## Results

**Figure 1:**
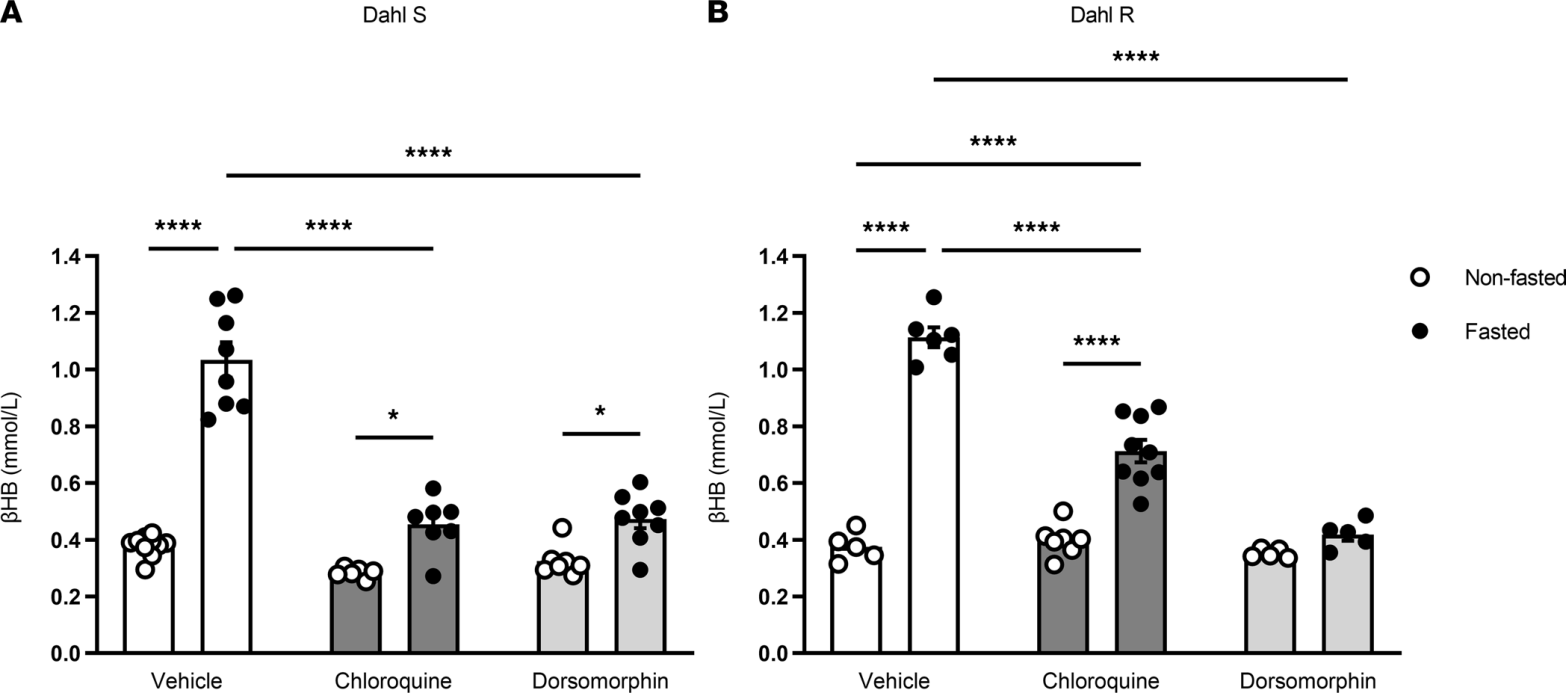
β-Hydroxybutyrate biosynthesis is autophagy dependent. β-Hydroxybutyrate (βHB) was measured in serum samples from nonfasted (free access to food) and fasted (24 hours) Dahl S (**A**) and Dahl R (**B**) rats (both low-salt diet fed), pretreated with and without lysosome acidification inhibitor chloroquine (2 × 50 mg/kg) or AMPKα inhibitor dorsomorphin (25 mg/kg). Mean ± SEM. *n* = 5–10. Two-way ANOVA: *P < 0.05, ****P < 0.0001.

Evolutionarily, autophagy serves to mobilize nutrients in times of starvation, including substrates for ketogenesis (11). Therefore, we hypothesized that inhibition of autophagy would prevent βHB biosynthesis in response to a 24-hour fast. Treatment with the lysosome acidification inhibitor chloroquine (CQ) impeded the starvation-induced increase in βHB in both Dahl S (Figure 1A) and Dahl salt–resistant (Dahl R) rats (Figure 1B). A major upstream regulator of starvation-induced autophagy is the energy sensor 5′ adenosine monophosphate–activated protein kinase α (AMPKα) Therefore, we hypothesized that inhibition of AMPKα would similarly prevent βHB generation after a 24-hour fast. Treatment with dorsomorphin, an ATP-competitive AMPKα inhibitor, also blocked the starvation-induced increase in βHB in both Dahl strains (Figure 1). These data suggest that βHB biosynthesis is dependent on AMPKα-induced autophagy.

**Figure 2:**
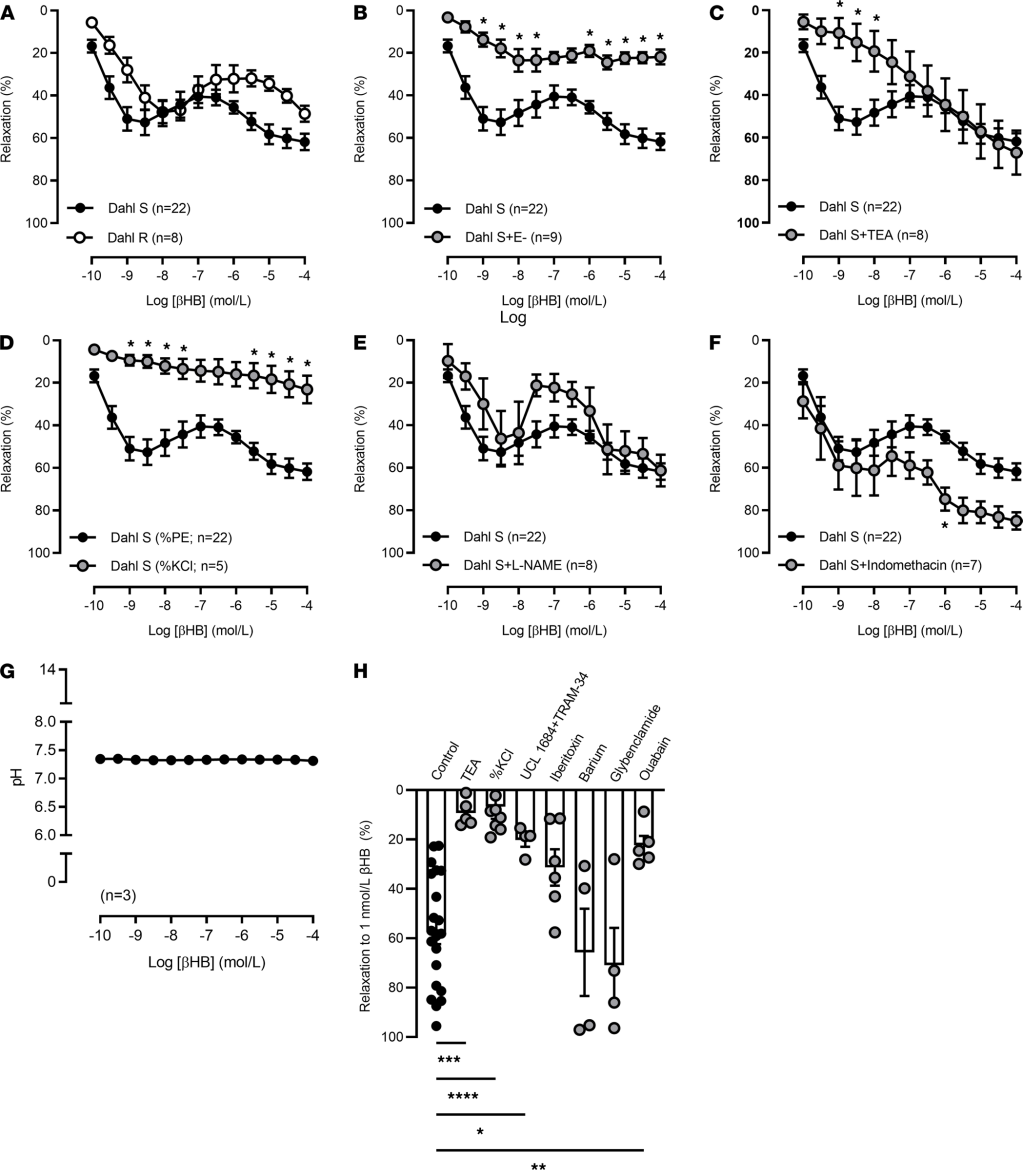
β-Hydroxybutyrate causes vasodilation via potassium channels and not nitric oxide biosynthesis or cyclooxygenase-derived products. Concentration-response curves to β-hydroxybutyrate (βHB) in mesenteric resistance arteries from low-salt diet–fed Dahl S and Dahl R rats (**A**). Some arteries from Dahl S rats were endothelium denuded (E-) (**B**); incubated with tetraethylammonium (TEA, 10 mmol/L) (**C**); contracted in response to potassium chloride (KCl, 120 mmol/L), as opposed to phenylephrine (10 μmol/L) (**D**); incubated with Nω-Nitro-L-arginine methyl ester (L-NAME, 100 μmol/L) (**E**); or incubated with indomethacin (10 μmol/L) (**F**). pH responses to βHB in Krebs buffer (**G**). Concentration-response curves to βHB in arteries from Dahl S rats were also incubated with UCL 1684 (100 nmol/L) and TRAM-34 (10 μmol/L) combined, iberiotoxin (100 nmol/L), barium chloride (100 μmol/L), glybenclamide (1 μmol/L), or ouabain (100 μmol/L) (**H**). The same control data are presented in multiple panels. n = 4–21. Mean ± SEM. Two-way ANOVA: *P < 0.05 (**B**–**D** and **F**). One-way ANOVA: *P < 0.05, **P < 0.01, ***P < 0.001, ****P < 0.0001; *t* test: *P < 0.05 (**H**).

Next, we questioned whether βHB could contribute to the beneficial effects of autophagy by exerting vasoactive effects. We have discovered for the first time to our knowledge that βHB is a potentially novel, liver-derived vasodilator. Specifically, when we administered βHB directly to isolated mesenteric resistance arteries from Dahl S and Dahl R rats, βHB caused potent relaxation (Figure 2A) but not contraction (Supplemental Figure 1; supplemental material available online with this article; https://doi.org/10.1172/jci.insight.149037DS1). Denudation of some arteries demonstrated that βHB is predominantly an endothelium-dependent vasodilator (Figure 2B and Supplemental Figure 2A), and inhibition of specific endothelium-derived vasodilators revealed that this relaxation was primarily mediated by potassium channels (Figure 2, C and D, and Supplemental Figure 2, B and C) and not by nitric oxide (Figure 2E and Supplemental Figure 2D), cyclooxygenase (Figure 2F and Supplemental Figure 2E), or changes in extracellular pH (Figure 2G). Additionally, this relaxation was not dependent on AMPKα, as incubation with dorsomorphin actually enhanced relaxation (Supplemental Figure 3, A and B). Investigation into specific potassium-dependent hyperpolarizing factors suggested that relaxation in response to βHB (1 nmol/L) was primarily mediated by small and intermediate conductance calcium-activated potassium channels and sodium/potassium ATPase, as relaxation was significantly attenuated by UCL 1684 and TRAM-34 (combined) and ouabain (Figure 2H). To support the potassium dependency of βHB-induced vasodilation, we also performed concentration-response curves in aortic segments. In physiological conditions, it is well established that vasodilation in the aorta occurs primarily through nitric oxide–dependent mechanisms (16). We observed minimal relaxation (6.1% ± 0.4%) in response to βHB in aorta from Dahl R rats, and there was no difference with and without nitric oxide inhibition (Supplemental Figure 4). Overall, these data reveal that βHB is a potent liver-derived vasodilator, predominately of resistance arteries.

**Figure 3:**
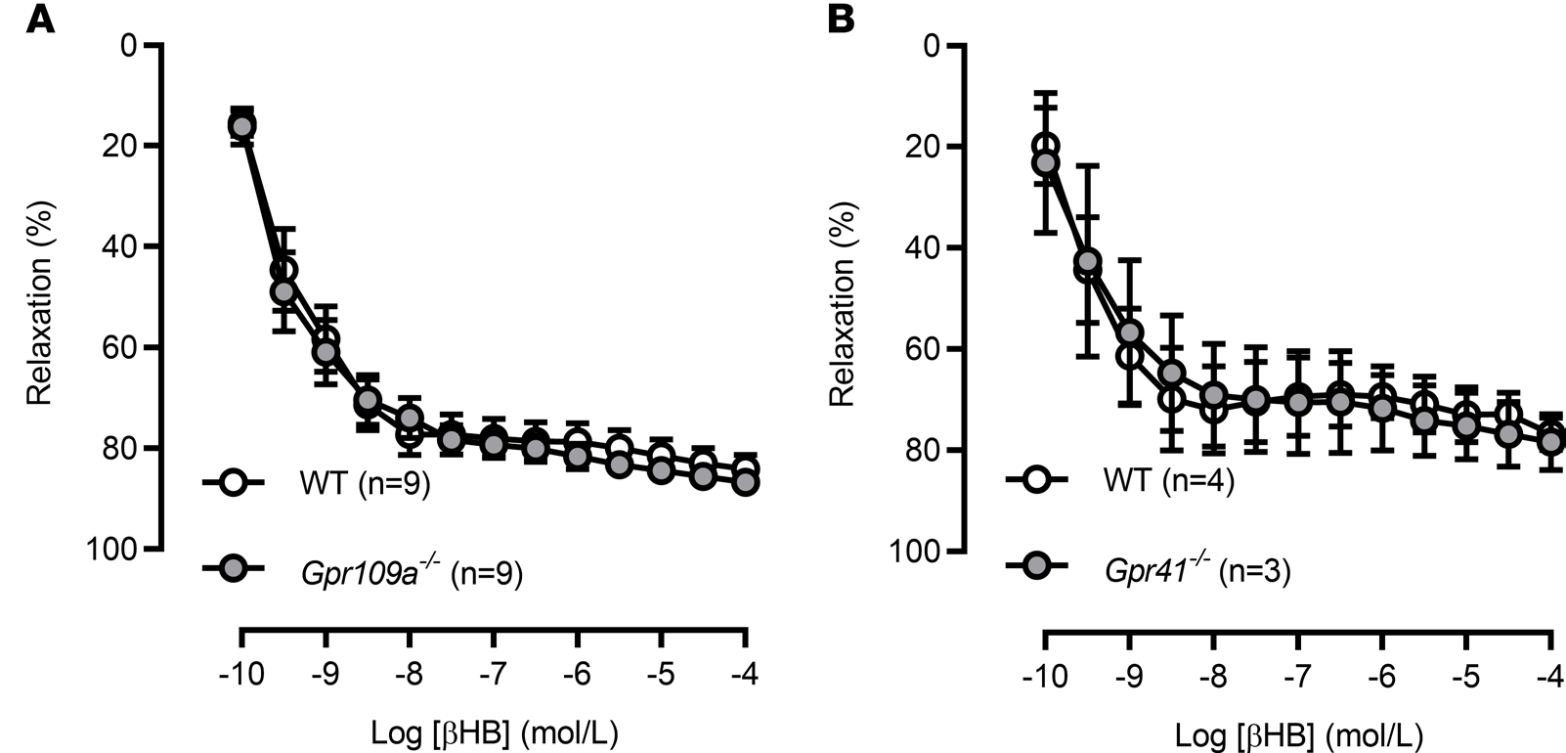
β-Hydroxybutyrate–induced vasodilation is not mediated via Gpr109a or Gpr41. β-Hydroxybutyrate (βHB) concentration-response curves in mesenteric resistance arteries from WT and *Gpr109a^–/–^* (**A**) and WT and *Gpr41^–/–^* (**B**) mice. Mean ± SEM. *n* = 3–9.

To further investigate the mechanism of βHB-induced relaxation, we needed to switch to a mouse model, as pharmacological antagonists for the classical βHB receptors Gpr109a and Gpr41 do not exist. Therefore, we obtained mice deficient in Gpr109a and Gpr41. As we expected, the vasodilation in response to βHB was conserved between rats and mice, but surprisingly, and, in opposition to our original hypothesis, there was no difference in the βHB-mediated vasodilation in mesenteric resistance arteries from *Gpr109a^–/–^* mice (Figure 3A) and *Gpr41^–/–^* mice (Figure 3B). These data reveal that βHB is causing vasodilation through a mechanism independent of its traditional GPCRs.

**Figure 4:**
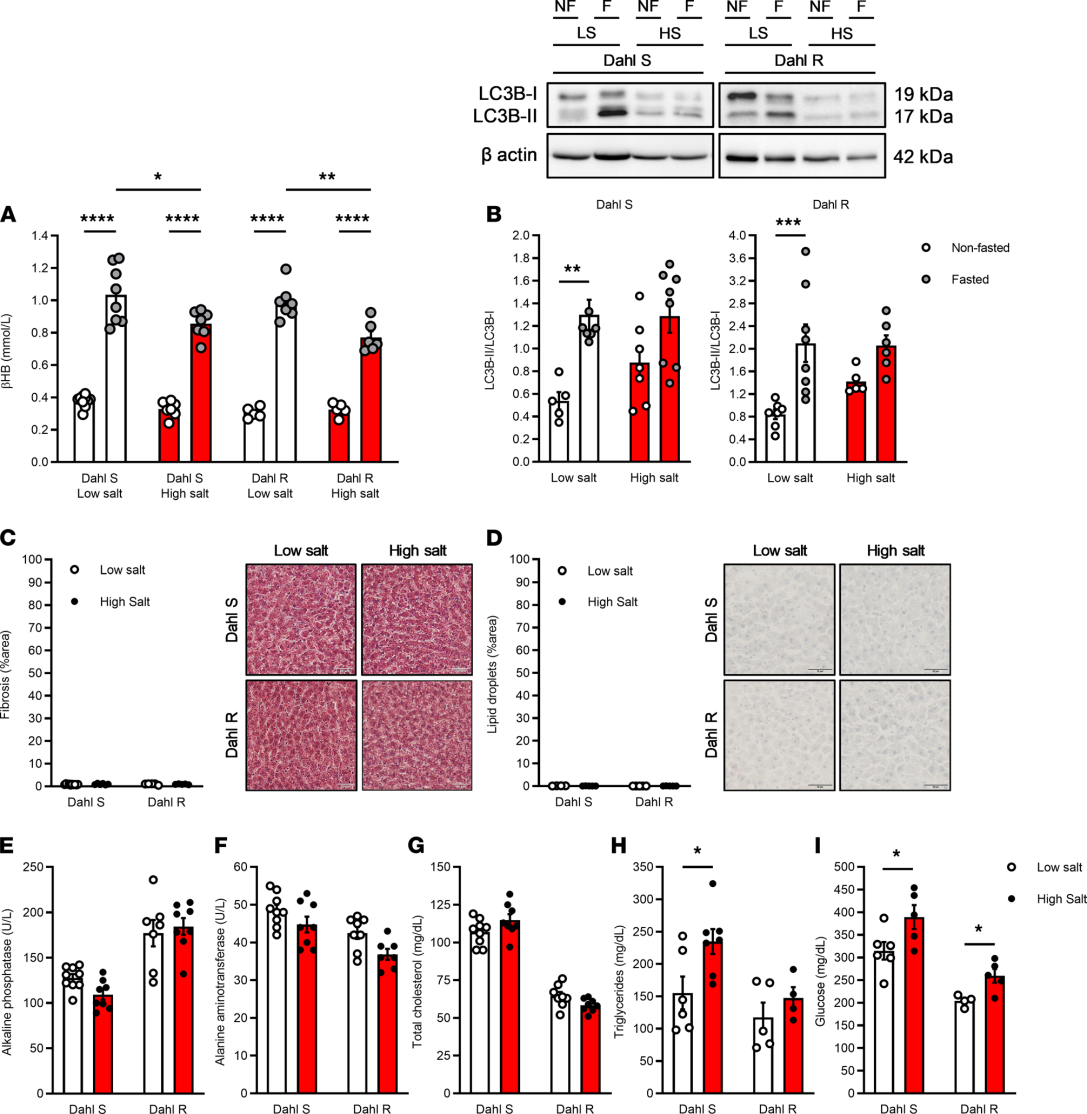
High-salt diet negatively regulates β-hydroxybutyrate synthesis and autophagic activity. β-Hydroxybutyrate (βHB) was measured in sera from nonfasted (free access to food) and fasted (24 hours) Dahl S and Dahl R rats that had consumed a low- or high-salt diet for 8 weeks (**A**). Protein expression analysis was performed for LC3B-II normalized to LC3B-I in liver biopsies from fasted and nonfasted low-salt diet– and high-salt diet–fed Dahl S and Dahl R rats (**B**). Representative images of immunoblots and densitometric analysis. Histological analysis was performed for fibrosis (**C**) and lipid droplets (**D**) in liver biopsies from nonfasted low-salt diet– and high-salt diet–fed Dahl S and Dahl R rats. Scale bar: 50 μm. Circulating liver enzymes (**E** and **F**), total cholesterol (**G**), triglycerides (**H**), and glucose (**I**) were measured in sera from nonfasted Dahl S and Dahl R rats that had consumed a low- or high-salt diet. Mean ± SEM. *n* = 4–10. Two-way ANOVA: *P < 0.05, **P < 0.01, ***P < 0.001, ****P < 0.0001 (**A** and **B**); *t* test: *P < 0.05 (**E**–**I**).

Previously, using an untargeted metabolomic approach, we reported that Dahl S rats fed a high-salt diet have less circulating βHB after a 24-hour fast compared with Dahl S rats fed a low-salt diet (14). In the current study, we have repeated these findings in Dahl S rats and observed the same phenomenon in normotensive Dahl R rats fed a high-salt diet (Figure 4A). This observation is important because it indicates that the diminished βHB biosynthesis after a 24-hour fast is not due to high blood pressure but actually due to the effects of a high-salt diet on the liver function. As a result, we hypothesized that a high-salt diet for 8 weeks would be associated with decreased autophagic activity in liver biopsies from Dahl S and Dahl R rats. We observed that, in liver biopsies from low-salt diet–fed Dahl S and Dahl R rats, there were increases in the ratio of microtubule-associated protein 1A/1B light chain 3B II (LC3B-II) to LC3B-I after a 24-hour fast, indicative of LC3B lipidation and enhanced autophagic activity. However, in high-salt diet–fed Dahl S and Dahl R rats, there was a lowered LC3B-II/LC3B-I ratio after a 24-hour fast (Figure 4B). See complete unedited blots in the supplemental material. In order to support this notion, we probed for the expression of p62 (also known as sequestosome 1). p62 is an autophagy substrate that can be used as a reporter of autophagic activity. Specifically, active autophagy leads to a decrease in p62 expression; conversely, inhibition of autophagy maintains p62 expression. We observed a significant decrease in p62 expression in liver biopsies from low-salt diet–fed Dahl S rats, and this decrease did not occur in high-salt diet–fed Dahl S rats (Supplemental Figure 5). Again, this supports the conclusion that a chronic high-salt diet leads to impaired autophagic activity. These changes in βHB and expression of representative autophagy proteins after a high-salt diet were not associated with liver fibrosis (Figure 4C), fatty liver (Figure 4D), traditional enzymatic markers of liver damage (Figure 4, E and F), or dysfunctional cholesterol metabolism (Figure 4G). On the other hand, nonfasting circulating triglycerides (Figure 4H) and glucose (Figure 4I) were higher in high-salt diet–fed Dahl S and Dahl R rats. Overall, these data suggest that a chronic high-salt diet decreases βHB biosynthesis and autophagy, possibly via elevated circulating energy substrates and not liver injury.

**Figure 5:**
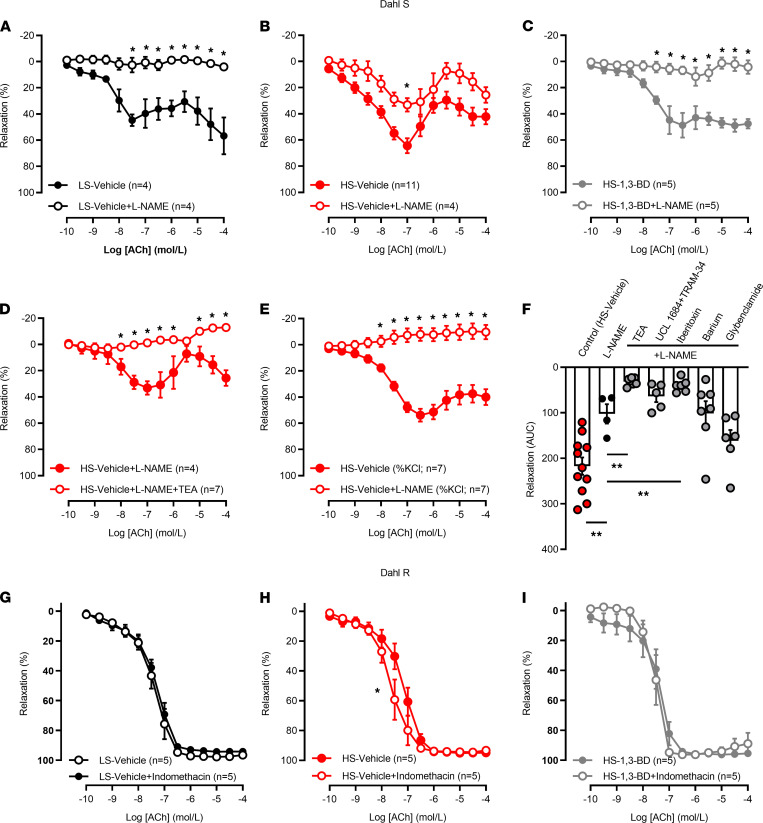
Reconstitution of β-hydroxybutyrate bioavailability prevents high-salt diet–induced endothelial dysfunction in Dahl S and Dahl R rats. Acetylcholine (ACh) concentration-response curves in mesenteric resistance arteries from low-salt diet–fed (LS-fed) Dahl S rats (**A**), high-salt diet–fed (HS-fed) Dahl S rats (**B**), and HS-fed animals Dahl S rats with 1,3-butanediol (1,3-BD; 20% v/v) (**C**) after incubation with Nω-Nitro-L-arginine methyl ester (L-NAME, 100 μmol/L). ACh concentration-response curves in L-NAME–incubated arteries from HS-fed Dahl S rats that were either coincubated with tetraethylammonium (TEA, 10 mmol/L) (**D**) or contracted in response to potassium chloride (KCl, 120 mmol/L), as opposed to phenylephrine (10 μmol/L) (**E**). ACh concentration-response curves in L-NAME–incubated arteries from HS-fed Dahl S rats that were also coincubated with UCL 1684 (100 nmol/L) and TRAM-34 (10 μmol/L) combined, iberiotoxin (100 nmol/L), barium chloride (100 μmol/L), or glybenclamide (1 μmol/L) (**F**). ACh concentration-response curves in arteries from LS-fed Dahl R rats (**G**), HS-fed Dahl R rats (**H**), and HS-fed Dahl R rats treated with 1,3-BD (**I**) after incubation with indomethacin (10 μmol/L). Mean ± SEM. *n* = 4–11. Two-way ANOVA: *P < 0.05 (**A**–**E**); 1-way ANOVA: **P < 0.01 (**F**); nonlinear regression (logEC_50_): *P < 0.05 (**H**).

To increase βHB bioavailability chronically, in vivo, and in lieu of nutrient deprivation, the exogenous 1,3-butanediol (1,3-BD) is commonly administered in drinking water as a metabolic precursor. After consumption, 1,3-BD is catabolized by the liver into βHB (17). Interestingly, when we administered 1,3-BD directly to isolated mesenteric resistance arteries from Dahl S and Dahl R rats, we observed a potent relaxation (Supplemental Figure 6), similar to what we observed with βHB. Despite this to our knowledge previously unrecognized duel function of 1,3-BD, we wanted to know what happens to vascular function if we increase βHB bioavailability in conjunction with a high-salt diet. To answer this question, we added 1,3-BD (20%) to the drinking water of Dahl S and Dahl R rats fed a high-salt diet for 8 weeks. Previously, we confirmed that this concentration is sufficient to significantly raise serum βHB bioavailability in high-salt diet–fed Dahl S rats (14). While the magnitude of endothelium-dependent relaxation in response to acetylcholine in mesenteric resistance arteries from Dahl S rats was similar between treatments, we observed that a high-salt diet and 1,3-BD changed the mechanism of endothelium-dependent vasodilation. Specifically, a high-salt diet shifted the relaxation away from a solely nitric oxide–dependent mechanism (Figure 5A) to a combination of nitric oxide– and nitric oxide–independent mechanisms (Figure 5B); 1,3-BD treatment prevented the shift in the vasodilator mechanism in response to a high-salt diet and primarily maintained a nitric oxide–dependent vasodilation (Figure 5C). Activation of potassium channels is a well-established compensatory mechanism to maintain vasodilation in conditions of low nitric oxide bioavailability (18). In support of this dogma, we observed that the relaxation in response to acetylcholine in mesenteric resistance arteries from high-salt diet–fed Dahl S rats could be completely blocked by nonspecific potassium channel blockade and Nω-Nitro-L-arginine methyl ester (L-NAME) (Figure 5, D and E), and application of specific inhibitors revealed that large-conductance calcium-activated potassium channels were the major potassium channel contributing to this relaxation in high-salt diet–fed Dahl S rats (Figure 5F). Overall, these data demonstrate that maintenance of βHB bioavailability prevents the shift away from nitric oxide–mediated vasodilation in Dahl S rats on a high-salt diet.

In Dahl R rats, we observed that a high-salt diet decreased the sensitivity of isolated mesenteric resistance arteries to acetylcholine, and 1,3-BD treatment prevented this endothelial dysfunction (logEC_50_, Dahl R–low salt, –7.35 ± 0.05 vs. Dahl R–high salt, –7.19 ± 0.07 [*P* < 0.05] vs. Dahl R–high salt + 1,3-BD, –7.42 ± 0.10). Furthermore, when mesenteric resistance arteries were incubated with the cyclooxygenase inhibitor indomethacin, only Dahl R rats on a high-salt diet presented with an increased sensitivity to acetylcholine (logEC_50_, high salt, –7.19 ± 0.07 vs. high salt + indomethacin, –7.66 ± 0.08; *P* < 0.05), indicating that 1,3-BD treatment decreased cyclooxygenase-induced vascular inflammation (logEC_50_, 1,3-BD, –7.42 ± 0.10 vs. 1,3-BD + indomethacin, –7.61 ± 0.09) (Figure 5, G–I). In summary, despite the strain differences in the mechanism of endothelial dysfunction after a high-salt diet, overall, these data indicate that maintenance of βHB bioavailability with 1,3-BD treatment prevents high-salt diet–induced endothelial dysfunction in Dahl S and Dahl R rats.

Interestingly, upon termination of the chronic treatment of 1,3-BD for 8 weeks, we observed that treated rats from both strains were significantly smaller than vehicle-treated rats (both low- and high-salt diets) (Supplemental Figure 7A). Further investigation revealed that total body mass (Supplemental Figure 7, B and E), epididymal fat mass (Supplemental Figure 7, C and F), and tibia length (Supplemental Figure 7, D and G) were all significantly smaller in 1,3-BD–treated rats. Analysis of systemic ion concentration revealed an increase in the anion gap in 1,3-BD–treated rats, a clinical measure of metabolic acidosis (Supplemental Figure 8, A and H); hepatotoxicity, as indicated by systemic increases in liver enzymes (Supplemental Figure 8, B, C, I, and J); bile acids (Supplemental Figure 8, D and K); bilirubin (Supplemental Figure 7, E and L); and hemoconcentration, as indicated by albumin (Supplemental Figure 8, F and M). Metabolically, we observed that 1,3-BD treatment significantly reduced nonfasting blood glucose and serum triglycerides to levels that were similar to those of rats that underwent a 24-hour fast (Supplemental Figure 7, B and E, and Supplemental Figure 9, A and D). On the other hand, 1,3-BD treatment significantly lowered circulating total cholesterol in Dahl S rats (Supplemental Figure 9C) but elevated it Dahl R rats (Supplemental Figure 9F). Collectively, these data suggest that 1,3-BD is a caloric restriction mimetic that improves cardiometabolic health in rats on a high-salt diet. On the other hand, it also has deleterious side effects at the dose used in the current study, including stunted growth, metabolic acidosis, and hepatotoxicity. Therefore, we acknowledge that the dose used in the current study is a limitation, and future work will focus on optimizing this dose for translational application.

## Discussion

Precisely how autophagy is vasculoprotective and ameliorates vascular damage is the focus of intense and rigorous research (4–6). Until now, most of the important discoveries surrounding autophagy and vascular physiology are centered on the premise that reduced autophagy leads to the accumulation of damaged cellular debris and dysfunctional organelles that causes inflammation and oxidative stress. Subsequently, this proinflammatory/prooxidative milieu quenches nitric oxide bioavailability (7, 8) and also uncouples endothelial nitric oxide synthase (eNOS) (19). Therefore, upregulation/reconstitution of autophagy decreases these vascular dysfunctions. While these rigorous studies have provided an important phenotypic understanding of how autophagy can ameliorate vascular function and structure, most of these studies have attempted to upregulate autophagy in the vasculature by using systemic interventions (e.g., lifestyle modifications or pharmacological agents administered orally). Therefore, these studies cannot rule out other organ systems contributing to the vascular phenotypes measured.

Here, we reveal for the first time to our knowledge that βHB is an autophagy-sensitive metabolite from the liver that can cause vasodilation via potassium channels. This work is paradigm shifting, as it offers an alternative mechanism to the antiinflammatory and antioxidative stress hypothesis of autophagy-induced vasculoprotection and it posits a reason as to why systemic interventions that elevate autophagy, such as intermittent fasting and exercise, are healthful. In this investigation, we also reveal for the first time to our knowledge that a high-salt diet decreases autophagy and offer one mechanism by which βHB is negatively regulated. While the precise mechanisms as to how high salt negatively regulates autophagic activity need to be confirmed, we suggest that this related with metabolic dysfunction and excess availability of energy substrates (i.e., hyperglycemia and hypertriglyceridemia), as opposed to overt liver damage. Regardless of the underlying mechanism, the decreased autophagy phenotype is translationally significant, as 90% of Americans exceed recommendations for salt consumption (20), and excess salt consumption is responsible for 1 of every 10 cardiovascular deaths (21). Moreover, our data reveal that the adverse effects of a high-salt diet on autophagy and βHB biosynthesis are independent of genetic susceptibility to salt-sensitive hypertension. Reconstituting βHB bioavailability, which would be analogous to chronic interventions that stimulate autophagy, prevented endothelial dysfunction caused by a high-salt diet. However, it should also be noted that 1,3-BD treatment also stunted growth and caused metabolic acidosis. Therefore, if 1,3-BD is to be promoted as a health-enhancing nutraceutical, the dosage needs to be titrated and optimized.

Previous studies investigating autophagy-dependent vasculoprotection have suggested that autophagy increases or maintains nitric oxide bioavailability by decreasing reactive oxygen species generation (7, 8), increasing eNOS coupling (19), preventing the pathological switch from nitric oxide– to hydrogen peroxide–mediated vasodilation (22), and increasing P2Y purinoceptor 1 receptor–mediated activation of eNOS (23). In contrast to these reports, we observed that βHB is a nitric oxide–independent vasodilator, as nitric oxide synthase inhibition did not prevent βHB-induced vasodilation, but potassium channel inhibitors did. In support of our findings, it is well established that the contribution of nitric oxide to vasodilation decreases in resistance arteries and arterioles, and the contribution of nitric oxide–independent mechanisms to vasodilation increases, particularly potassium efflux (sometimes referred to as endothelium-derived hyperpolarizing factor [EDH]), in cardiovascular diseases such as hypertension (16). Our current data are consistent with those of our previous report, in which we systemically administered the mTOR-independent autophagy activator trehalose and observed an improved endothelium-dependent relaxation via enhanced potassium-induced relaxation also in mesenteric resistance arteries (9).

Classically, βHB production is controlled by at least 2 nutrient-responsive pathways that are implicated in longevity (mTORC1 and FOXA2) and may be subject to regulation by βHB via histone deacetylase inhibition (15). Additionally, βHB can also signal via 2 known GPCRs. βHB can activate Gpr109a, a high-affinity G_i/o_-GPCR (24), and is generally recognized as an antagonist of Gpr41, another G_i/o_-GPCR (25). However, there are conflicting reports on whether βHB functions as an antagonist or agonist for Gpr41 (26). In line with this, we hypothesized that βHB caused potent vasodilation via Gpr109a activation. To our surprise, mesenteric resistance arteries from mice deficient in Gpr109a still relaxed in response to βHB, as did arteries from Gpr41-deficient mice. Therefore, the mechanism by which βHB causes vasodilation still remains to be confirmed. An interesting phenomenon that was observed throughout our study was the biphasic response of βHB-induced vasodilation in mesenteric resistance arteries from Dahl S and Dahl R rats. Although the potent βHB-induced vasodilation of mesenteric resistance arteries appears conserved across rodent species, arteries from Dahl rats had a unique contractile response after the initial vasodilation (~1–10 nmol/L). This contraction was not observed in mice (Figure 3) or rats from the Wistar background (data not shown). This is a perplexing phenomenon, as βHB does not cause contraction in arteries at basal tone or in those minimally contracted with 26.2 mmol/L potassium chloride (KCl), which opens calcium channels (Supplemental Figure 1). Therefore, the contractile response appears to be dependent on the initial vasodilation/hyperpolarization, and it is not due to cyclooxygenase. Overall, the etiology of this biphasic response in Dahl rats still remains to be elucidated.

Furthermore, the vasodilation of isolated mesenteric resistance arteries in response to 1,3-BD, classically employed as a βHB precursor, has important consideration for studies that aim to reconstitute βHB bioavailability. Our results pharmacologically redefine 1,3-BD from solely a βHB precursor (17) to potentially a βHB mimetic as well. Therefore, this duel action of 1,3-BD needs to be taken into consideration when interpreting studies (including ours) that seek to increase endogenous βHB bioavailability by administering 1,3-BD, as 1,3-BD has direct vasodilatory actions that are independent of its metabolism into βHB.

In summary, we have shown for the first time to our knowledge that βHB is an autophagy-dependent liver-derived relaxing factor that has direct vasodilating actions on the vasculature and that a high-salt diet can decrease its biosynthesis by interfering with autophagy. Therefore, this work reveals both a potentially novel mechanism by which autophagy is vasculoprotective and a pathogenic mechanism of decreased autophagic activity and vascular deterioration. Furthermore, these data suggest a health-promoting mechanism underlying interventions that raise ketone bodies (e.g., intermittent fasting or ketogenic diets). Our results illustrate that βHB, the most physiologically abundant ketone body, can have direct vasodilatory actions. Therefore, enhanced vascular health after interventions such as intermittent fasting and ketogenic diets are not necessarily only a secondary effect of improvements in metabolic indices (e.g., glucose control and/or insulin sensitivity).

## Methods

### Experimental animals.

The inbred Dahl S (SS/Jr) and Dahl R (SR/Jr) rat strains were used. Dahl S and Dahl R rats are widely used preclinical models for genetic susceptibility and resistance to hypertension induced by a high-salt diet, respectively (27, 28). The inbred strains of the Dahl S and Dahl R rat models were developed in our laboratory at the University of Toledo College of Medicine and Life Sciences (previous Medical College of Ohio) and have been maintained in-house since 1985 (29, 30). Due to the inherent propensity of the Dahl S strain to develop hypertension, both Dahl strains were bred and maintained on a low-salt diet (0.3% NaCl; Teklad diet 7034, Envigo). For experiments involving a high-salt diet, 2% NaCl was used (Teklad diet TD.94217).

In some experiments, due to the lack of pharmacological antagonists and mutant rats, mice genetically deficient in Gpr109a and Gpr41 were used. Mice lacking Gpr109a were used with permission from Stefan Offermanns (Max Planck Institute for Heart and Lung Research, Bad Nauheim, Germany), and mice lacking Gpr41 were used with permission from Masashi Yanagisawa (University of Tsukuba, Tsukuba, Japan). Both mouse strains were backcrossed onto a C57BL/6J background, and heterozygous mice were intercrossed for at least 4 generations. Genotyping for Gpr109a deficiency was performed using the PUMA-G sense-1 primer 5′-TCAGATCTGACTCGTCCACC-3′ in combination with either 5′-CCTCTTCGCTATTACGCCAGC-3′ for the inactivated Gpr109a allele or 5′-CCATTGCCCAGGAGTCCGAAC-3′ for the WT allele (31). Genotyping for Gpr41 deficiency used the primer set 5′-CACACTGCTCGATCCGGAACCCTT and 5′-GAGAACTGTCTGGAAAACGCTCAC for the inactivated Gpr41 allele and 5′-CGACGCCCAGTGGCTGTGGACTTA and 5′-GTACCACAGTGGATAGGCCACGC for the WT allele (32). All mice were bred in-house and maintained on the 2916 Teklad global 16% protein diet.

All rodent pups were weaned between 28 and 30 days. For most experimental procedures, rats were at least 12 weeks of age; untreated rats used for the acute vascular function experiments in response to βHB were 7–8 weeks old. Mice were at least 10 weeks of age when used. Only male rodents were used in the current studies. All rodents were maintained on a 12:12-hour-light/dark cycle and were allowed access to both chow and water ad libitum, unless specifically fasted (see *Treatments* below). Euthanasia of rodents was performed by thoracotomy, and exsanguination via cardiac puncture was performed under isoflurane anesthesia, administered via nose cone (5% in 100% O_2_), consistent with the 2013 American Veterinary Medical Association Guidelines for the Euthanasia of Animals. All euthanasia and tissue harvesting were performed in the Department of Laboratory Animal Resources from 0900 to 1100 on experimental days.

The sample size per experiment (see figure panels and legends) is the number of independent rodents used, respective of strain and treatment group. Previous work from our laboratory estimating a large effect size (Cohen’s *d* > 0.8), as well as power analysis (desired power of 0.80 to 0.85 with a probability of a type I error of 0.05), has provided a basis for the projected number of rodents required per experimental group.

### Treatments.

To understand the mechanisms of endogenous βHB biosynthesis, Dahl S and Dahl R rats of at least 12 weeks of age were randomly assigned to a 24-hour fast or allowed continued access to the low-salt chow (nonfasted). Both groups continued to have free access to drinking water. Some nonfasted and fasted rats were also randomized to receive intraperitoneal injections of lysosome acidification inhibitor CQ (50 mg/kg) (MilliporeSigma), or AMPKα inhibitor, dorsomorphin (25 mg/kg) (MedChemExpress), immediately prior to food withdrawal. In the case of CQ, a second dose was administered after 12 hour. Both drugs were prepared in saline (0.9% NaCl, vehicle).

To understand the consequences of a high-salt diet on hepatic autophagy and βHB biosynthesis, Dahl S and Dahl R rats were switched to the high-salt diet at 5 weeks of age and maintained on this diet until 13 weeks of age. To understand if reconstitution of βHB bioavailability could prevent the vascular damage induced by high-salt diet, groups of Dahl S and Dahl R rats were administered 1,3-BD (MilliporeSigma) in their drinking water (20% v/v), as we have performed previously (14).

### βHB measurement.

Under isoflurane anesthesia, but prior to thoracotomy and exsanguination via cardiac puncture, arterial blood was collected from the abdominal aorta in silicone-coated collection tubes specified for serum (BD Vacutainer). Blood was left to clot at room temperature for approximately 20 minutes. After clotting, blood was centrifuged at 2000*g* for 15 minutes at 4°C, and the serum was separated, collected, flash frozen in liquid nitrogen, and stored at –80°C until the time of measurement. βHB measurement was performed using a colorimetric assay according to the manufacturer’s instructions (Cayman Chemical).

### Vascular function.

Third-order mesenteric resistance arteries (rats) and second-order mesenteric resistance arteries (mice) were mounted onto Danish Myo Technology (DMT) wire myographs and thoracic aortic segments (rats) were mounted onto DMT pin myographs. All arteries and segments were bathed in 37°C Krebs buffer with 5% CO_2_ and 95% O_2_ throughout the experiment. Mesenteric resistance arteries were normalized to a lumen diameter optimal for tension development and aortic segments were set to a passive force of 30 mN and allowed to stabilize for 30 minutes, as described previously and recommended (33, 34). All arteries and segments were initially contracted with KCl (120 mmol/L). Endothelium integrity was then tested with a phenylephrine-induced contraction (3 μmol/L) followed by endothelium-dependent vasodilation with acetylcholine (3 μmol/L).

Cumulative concentration-response curves were performed in response to acetylcholine (0.1 nmol/L to 100 μmol/L) (MilliporeSigma), βHB (0.1 nmol/L to 100 μmol/L) (Cayman Chemical), and 1,3-BD (0.1 nmol/L to 100 μmol/L). Acetylcholine and 1,3-BD were prepared in deionized water and βHB in dimethyl sulfoxide. All relaxation concentration-response curves were performed after an initial contraction with phenylephrine (10 μmol/L) or KCl (120 mmol/L).

To understand the contribution of the endothelium and endothelium-derived factors, some arteries and segments were denuded with a hair shaft immediately after mounting or they were incubated with different pharmacological inhibitors for 30 minutes immediately prior to acetylcholine and βHB concentration-response curves. Specific inhibitors included tetraethylammonium (10 mmol/L), L-NAME (100 μmol/L), indomethacin (10 μmol/L), dorsomorphin (10 μmol/L), UCL 1684 (100 nmol/L) and TRAM-34 (10 μmol/L) combined, iberiotoxin (100 nmol/L), barium chloride (100 μmol/L), glybenclamide (1 μmol/L), or ouabain (100 μmol/L). All inhibitors for vascular function experiments were purchased from MilliporeSigma. Relaxation concentration-response curves are presented as a percentage of the contractile response, and contraction concentration-response curves are presented in units of absolute force (mN).

### Western blotting.

After euthanasia, liver biopsies from the left anterior lobe were immediately flash frozen in liquid nitrogen. Frozen biopsies were then homogenized using a mortar and pestle and lysed using tissue protein extraction reagent (Thermo Fisher Scientific), with protease inhibitors (sodium orthovanadate, phenylmethylsulfonyl fluoride, and protease inhibitor cocktail) and phosphatase inhibitors (sodium fluoride and sodium pyrophosphate) (all MilliporeSigma). Protein concentration of liver lysates was subsequently determined, and then equal quantities of protein (60 μg) were loaded into 12% polyacrylamide gels. After loading, gels were separated by sodium dodecyl sulfate polyacrylamide gel electrophoresis and transferred to Amersham Protran nitrocellulose membranes (GE Healthcare). Protein expression for LC3B-I and -II (1:1000) (NB600-1384, Novus Biologicals, sourced from rabbit) and p62 (1:1000) (Cell Signaling Technology, 23214, sourced from rabbit) was determined; β actin (1:5000) (A3854, MilliporeSigma, purified from hybridoma cell culture) was used as the loading control. Densitometric analysis was performed by ImageJ (NIH).

### Histology.

Masson’s trichrome staining was used to measure fibrosis. Liver biopsies from the left anterior lobe were immediately fixed 10% neutral buffered formalin (Thermo Fisher Scientific) for 24 hour and subsequently stored in ethanol (70%). Immediately prior to staining, biopsies were embedded in paraffin, and transverse cross sections (5 μm) were subsequently cut and processed by the University of Toledo College of Medicine and Life Sciences Histology Core following standard staining procedures. Stained cross sections were viewed with a light microscope (Olympus VS120) using a ×20 objective. Four different cross sections of each biopsy were examined for fibrosis, as indicated by the abundance of blue collagen staining. Images were analyzed using ImageJ.

Oil Red O staining was used to measure lipid droplet accumulation. Liver biopsies from the left anterior lobe were immediately embedded in tissue freezing medium and frozen in liquid nitrogen. Transverse cross sections (8 μm) were processed by the University of Toledo College of Medicine and Life Sciences Histology Core following standard staining procedures. Stained cross sections were viewed with a light microscope (Olympus VS120) using a ×20 objective. Four different cross sections of each biopsy were examined for the abundance and size of red lipid droplets. Images were analyzed using ImageJ.

### Blood chemistry analysis.

Circulating indicators of liver health, electrolytes, and glucose were measured with the VetScan VS2 Chemistry Analyzer (Zoetis). Using stored serum samples, the VetScan Mammalian Liver Profile reagent rotor was used to measure alkaline phosphatase, alanine aminotransferase, bile acids, bilirubin, albumin, and total cholesterol, and the VetScan Kidney Profile reagent rotor was used to measure chloride (Cl^–^), potassium (K^+^), sodium (Na^+^), total carbon dioxide (tCO_2_), and glucose. Metabolic acidosis was measured by calculating the anion gap: *Anion gap = Na^+^ —* (*Cl^–^ + (tCO_2_ — 1*)). Serum triglycerides were measured using a colorimetric assay according to the manufacturer’s instructions (Randox Laboratories).

### Statistics.

The statistical procedures used included 2-tailed Student’s *t* test, 1- and 2-way ANOVA, and nonlinear regression analysis (logEC_50_). Dunnett’s post hoc testing was used in all cases using a 1-way ANOVA, and the Šidák post hoc** was used in all cases using a 2-way ANOVA. All analyses were performed using GraphPad Prism 9.1.2 data analysis software. Statistical significance was set at *P* < 0.05. The data are presented as mean ± SEM.

### Study approval.

All breeding and experimental procedures were performed in accordance with the *Guide for the Care and Use of Laboratory Animals* (National Academies Press, 2011) and were reviewed and approved by the Institutional Animal Care and Use Committee of the University of Toledo College of Medicine and Life Sciences.

## Author contributions

CGM, SC, MVK, CFW, and BJ researched the concept and designed the study. CGM, SC, GS, BSY, ZJS, AS, BM, NRB, TY, and XC acquired data. CGM provided data analysis and interpretation. CGM drafted the manuscript. All authors reviewed and approved the submission. All authors are accountable for their contributions.

## Supplementary Material

Supplemental data

## Version 1 09/09/2021

In-Press Preview

## Version 2 10/22/2021

Electronic publication
