# Supplementary material for: Ketone body β-hydroxybutyrate is an autophagy-dependent vasodilator
Source: JCI Insight. 2021 Oct 22;6(20):e149037. doi: 10.1172/jci.insight.149037 (PMC8564907; doi:10.1172/jci.insight.149037)

Ketone body β-hydroxybutyrate is an autophagy-dependent vasodilator

Cameron G. McCarthy*, Saroj Chakraborty, Gagandeep Singh, Beng San Yeoh, Zachary J. Schreckenberger, Avinash Singh, Blair Mell, Nicole R. Bearss, Tao Yang, Xi Cheng, Matam Vijay-Kumar, Camilla F. Wenceslau, & Bina Joe*

Center for Hypertension & Personalized Medicine, Department of Physiology & Pharmacology, University of Toledo College of Medicine and Life Sciences, Toledo, OH, USA

***Corresponding authors:**
Cameron G. McCarthy and Bina Joe
University of Toledo College of Medicine and Life Sciences
Department of Physiology and Pharmacology
Center for Hypertension and Precision Medicine
Block Health Science Building
3000 Arlington Ave, Toledo, OH 43614-2598
Phone: (419) 383-4137
Emails: cameron.mccarthy@utoledo.edu and bina.joe@utoledo.edu

Conflict of interest statement
The authors have declared that no conflict of interest exists.

SUPPLEMENTARY FIGURES AND LEGENDS
Supplementary figure 1.

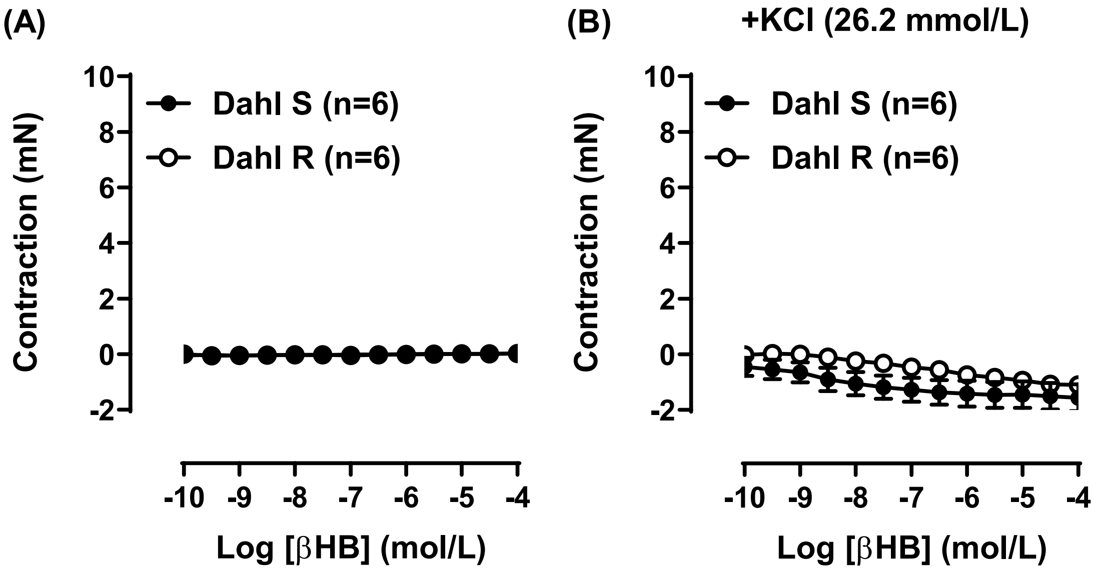

Supplementary figure 1. β-hydroxybutyrate (βHB) does not cause vascular contraction. Contraction concentration-response curves to βHB in mesenteric resistance arteries from Dahl S and Dahl R rats with basal tone (A) and after contraction to a low depolarizing concentration of potassium chloride (KCl, 26.2 mmol/L) (B). Mean ± SEM. n=6.

Supplementary figure 2.

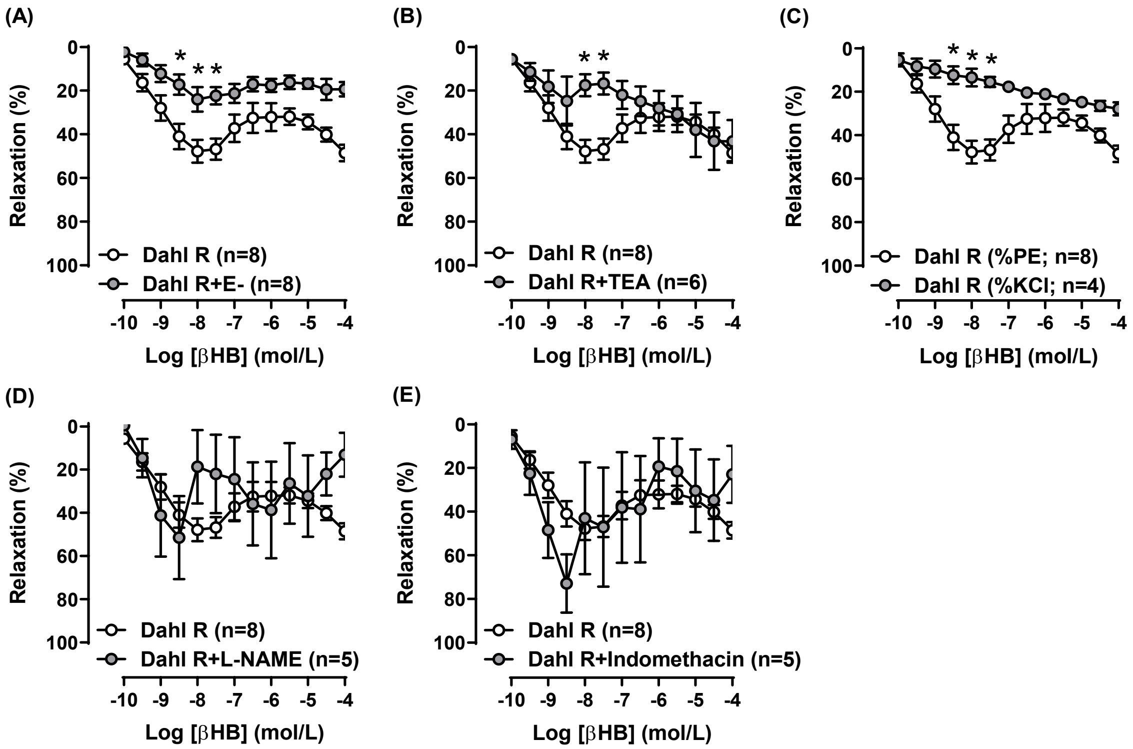

Supplementary figure 2. Concentration-response curves to β-hydroxybutyrate (βHB) in mesenteric resistance arteries from Dahl R rats. Some arteries were endothelium denuded (E-) (A), incubated with tetraethylammonium (TEA, 10 mmol/L) (B), contracted to potassium chloride (KCl, 120 mmol/L), as opposed to phenylephrine (10 μmol/L) (C), incubated with Nω-Nitro-L-arginine methyl ester (L-NAME, 100 μmol/L) (D), or incubated with indomethacin (10 μmol/L) (E). Mean ± SEM. n=4-8. Two-way ANOVA: *$p<0.05$.

Supplementary figure 3.

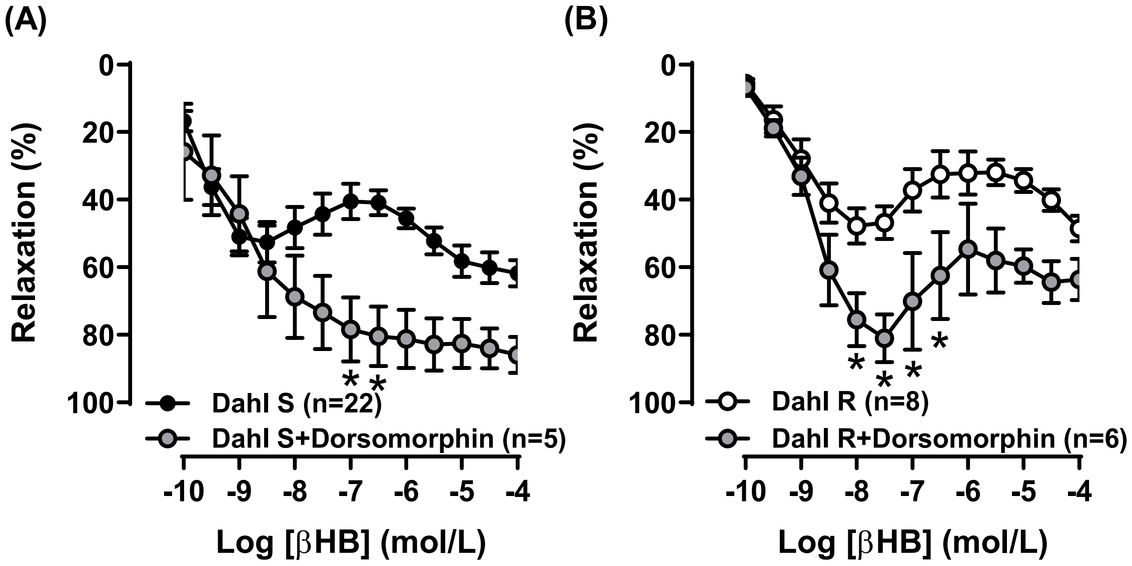

Supplementary figure 3. β-hydroxybutyrate (βHB)-dependent vasodilation is not mediated by AMPKα. Concentration-response curves to βHB in mesenteric resistance arteries from Dahl S (A) and Dahl R (B) rats, with and without the AMPKα inhibitor dorsomorphin (10 µmol/L). Mean ± SEM. n=5-21. Two-way ANOVA: *p<0.05.

Supplementary figure 4.

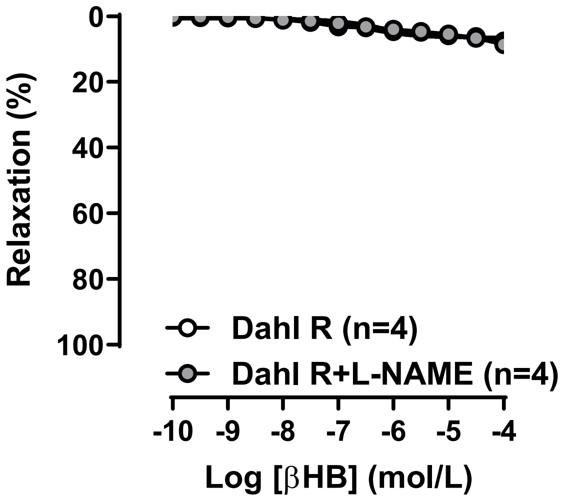

Supplementary figure 4. β-hydroxybutyrate (βHB)-dependent causes minimal nitric oxide-independent vasodilation in aorta. Concentration-response curves to βHB in aortic segments from Dahl R rats, with and without Nω-Nitro-L-arginine methyl ester (L-NAME, 100 µmol/L). Mean ± SEM. n=4.

Supplementary figure 5.

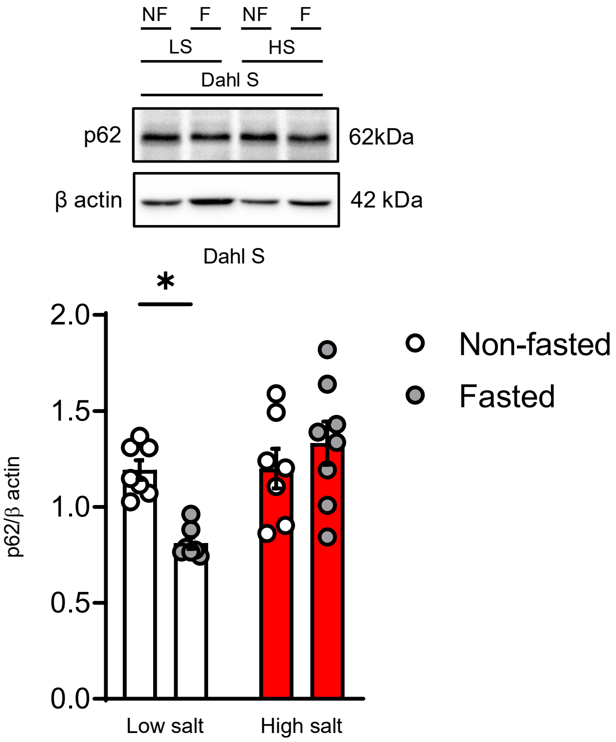

Supplementary figure 5. High salt diet negatively regulates autophagic activity. Protein expression analysis was performed for p62 normalized to β actin in liver biopsies from non-fasted (free access to food) and fasted (24 h) Dahl S rats that had consumed a low or high salt diet for 8 weeks. *Above*, representative images of immunoblots; *below*, densitometric analysis. Mean ± SEM. n=7-8. Two-way ANOVA: *$p<0.05$.

Supplementary figure 6.

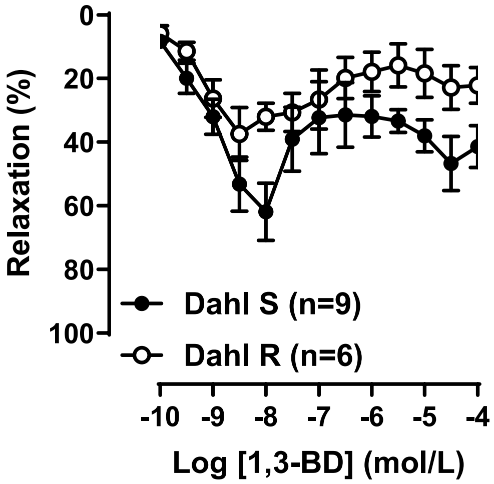

Supplementary figure 6. 1,3-Butanediol (1,3-BD) causes potent vasodilation, similar to β-hydroxybutyrate (βHB). Concentration-response curves to 1,3-BD in mesenteric resistance arteries from Dahl S (A) and Dahl R (B) rats. Mean ± SEM. n=6-9. Two-way ANOVA: *$p<0.05$.

Supplementary figure 7.

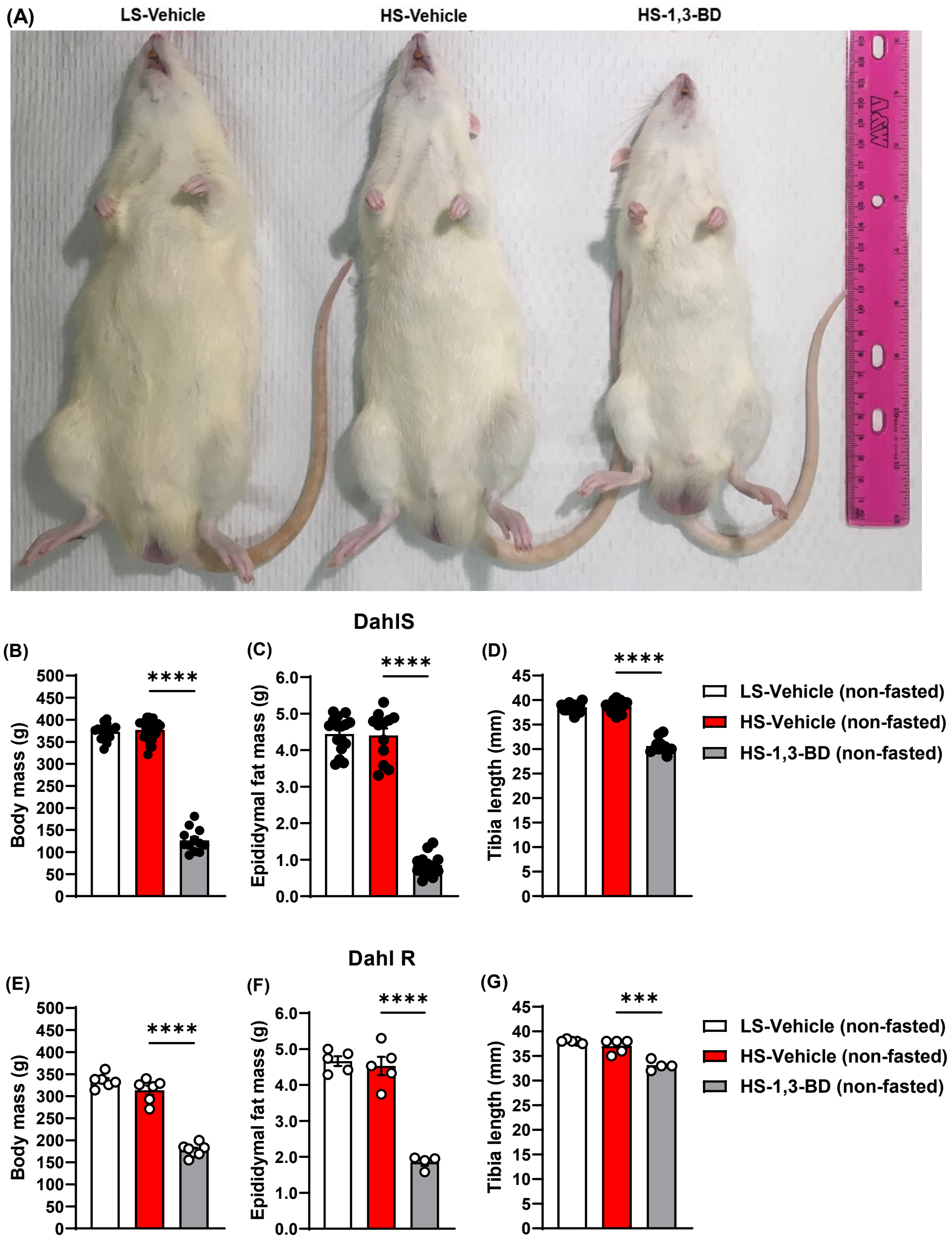

Supplementary figure 7. Reconstitution of β-hydroxybutyrate (βHB) bioavailability with 1,3-butanediol (1,3-BD) stunts growth. Dahl S and Dahl R rats were treated with either a low salt (LS) diet, a high salt (HS) diet, or a HS diet in conjunction with 1,3-BD (20% *v/v*) for eight weeks. Pictured are Dahl R rats after treatment (A). Body mass (B and E), epididymal fat mass (C and F), and tibia length (D and G) were measured in Dahl S and Dahl R rats, respectively. Mean ± SEM. n=4-28. One-way ANOVA: ***$p<0.001$, ****$p<0.0001$.

Supplementary figure 8.

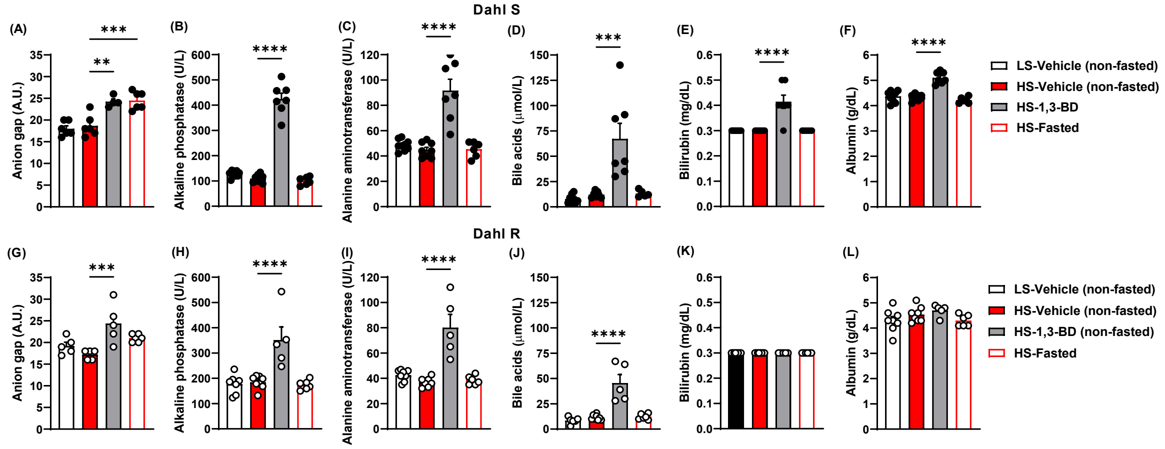

Supplementary figure 8. 1,3-Butanediol (1,3-BD) treatment causes metabolic acidosis and hepatotoxicity. Dahl S and Dahl R rats were treated with either a low salt (LS) diet, a high salt (HS) diet, or a HS diet in conjunction with 1,3-BD (20% *v/v*) for eight weeks. Some HS-fed rats from both strains were fasted for 24 h as a positive control. The anion gap (A and G), liver enzymes alkaline phosphatase (B and H) and alanine aminotransferase (C and I), bile acids (D and J), bilirubin (E and K), and albumin (F and L) were measured in serum from Dahl S and Dahl R rats, respectively. Mean ± SEM. n=4-10. One-way ANOVA: **p<0.01, ***p<0.001, ****p<0.0001.

Supplementary figure 9.

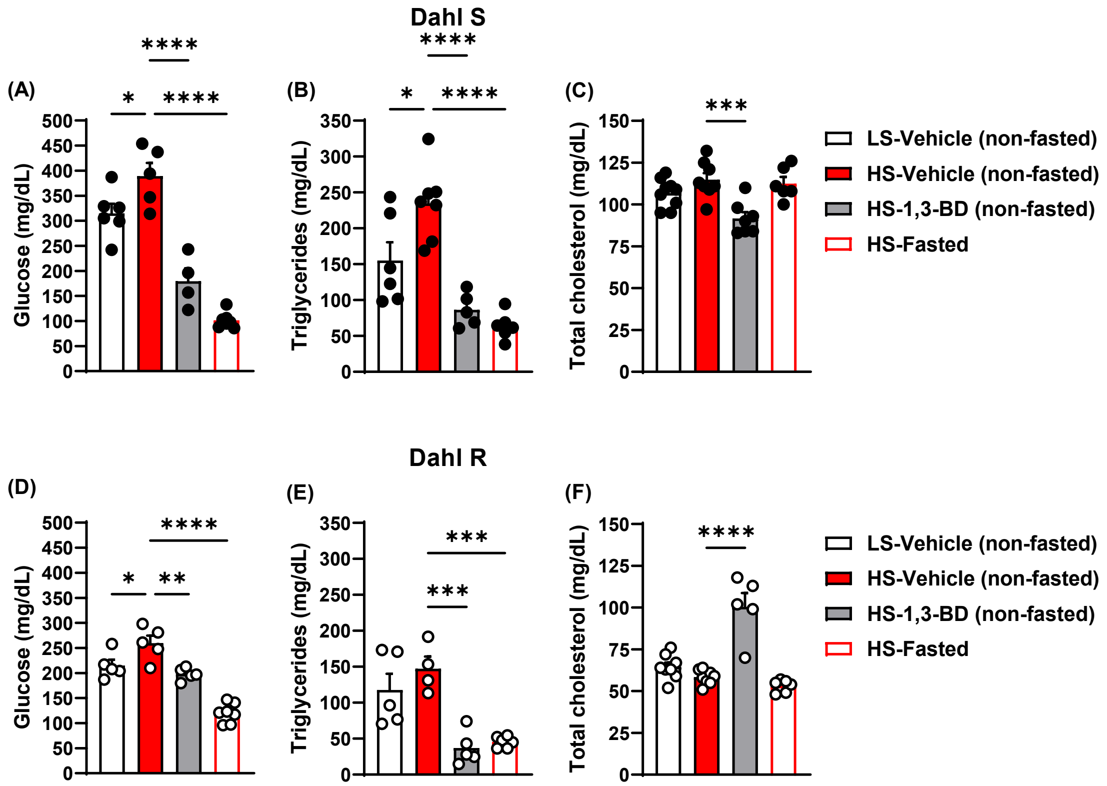

Supplementary figure 9. 1,3-Butanediol (1,3-BD) is a caloric restriction mimetic. Dahl S and Dahl R rats were treated with either a low salt (LS) diet, a high salt (HS) diet, or a HS diet in conjunction with 1,3-BD (20% *v/v*) for eight weeks. Some HS-fed rats from both strains were fasted for 24 h as a positive control. Circulating glucose (A and D), triglycerides (B and E), and total cholesterol (C and F) were measured in serum from Dahl S and Dahl R rats, respectively. Mean ± SEM. n=4-9. One-way ANOVA: *$p<0.05$, **$p<0.01$, ***$p<0.001$, ****$p<0.0001$.

Ketone body β-hydroxybutyrate is an autophagy-dependent vasodilator

Uncropped Western bots

McCarthy *et al.*

Submission to JCI Insights

How to read the uncropped/unrotated membranes: Set 1 loading

NF: Non-fasting
F: Fasting
LS: Low-salt
HS: High salt

F	NF	F	NF	F	NF	F	NF
HS		LS		HS		LS	
Dahl S sample 1				Dahl R sample 1			

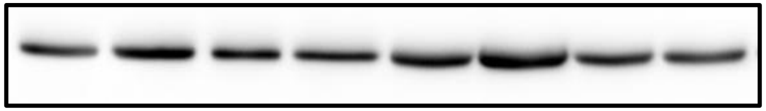

LADDER

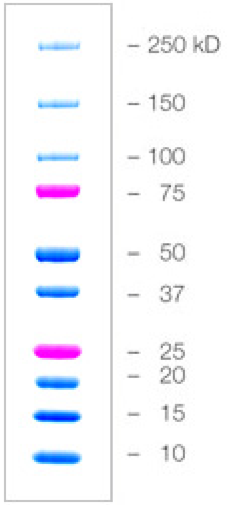

161-0374, Bio-rad (15 µl)

***Dahl S and Dahl R were analyzed independently**
****Gel 6 consisted of two sets of Dahl S samples, and no Dahl R**

Hepatic LC3B-I and -II ~19 and 17 kDa

Gel 1

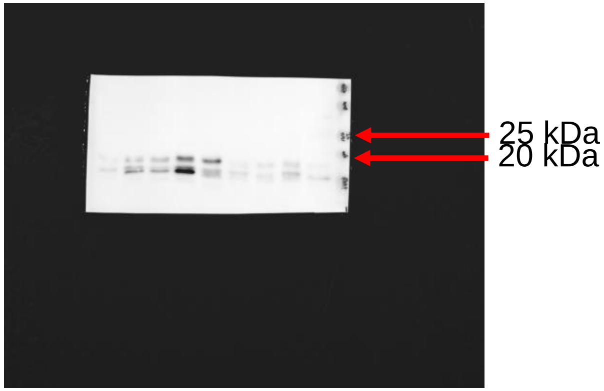

Gel 2

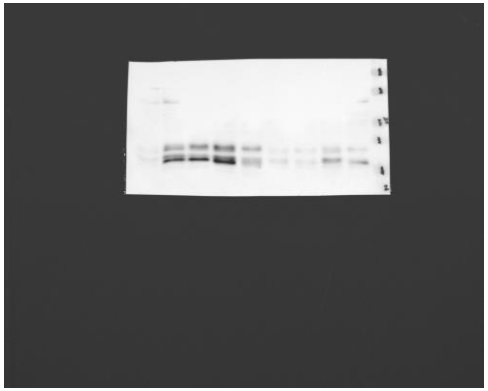

Gel 3

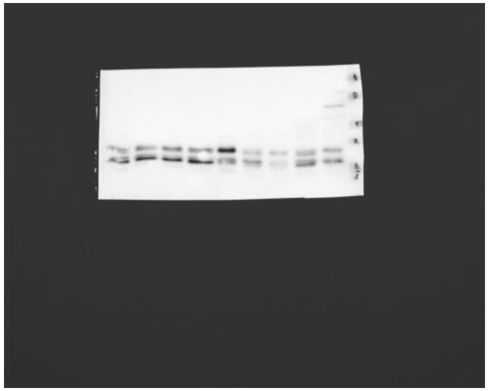

Gel 4

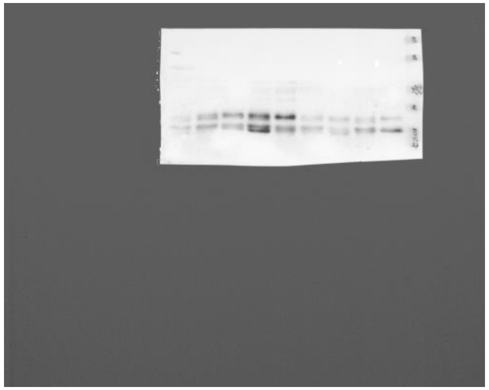

Gel 5

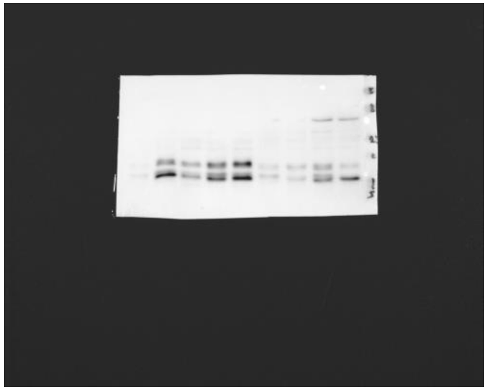

Gel 6

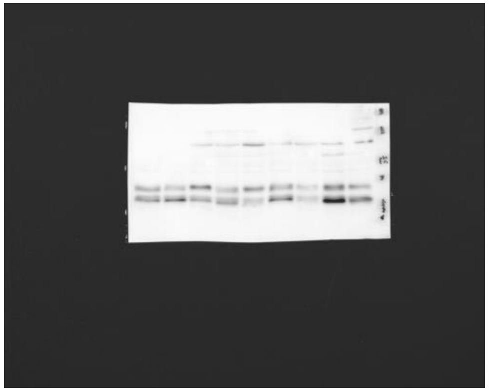

Gels: 12% acrylamide

NB600-1384, Novus Biologicals (1:1000)

Hepatic β actin – 42 kDa

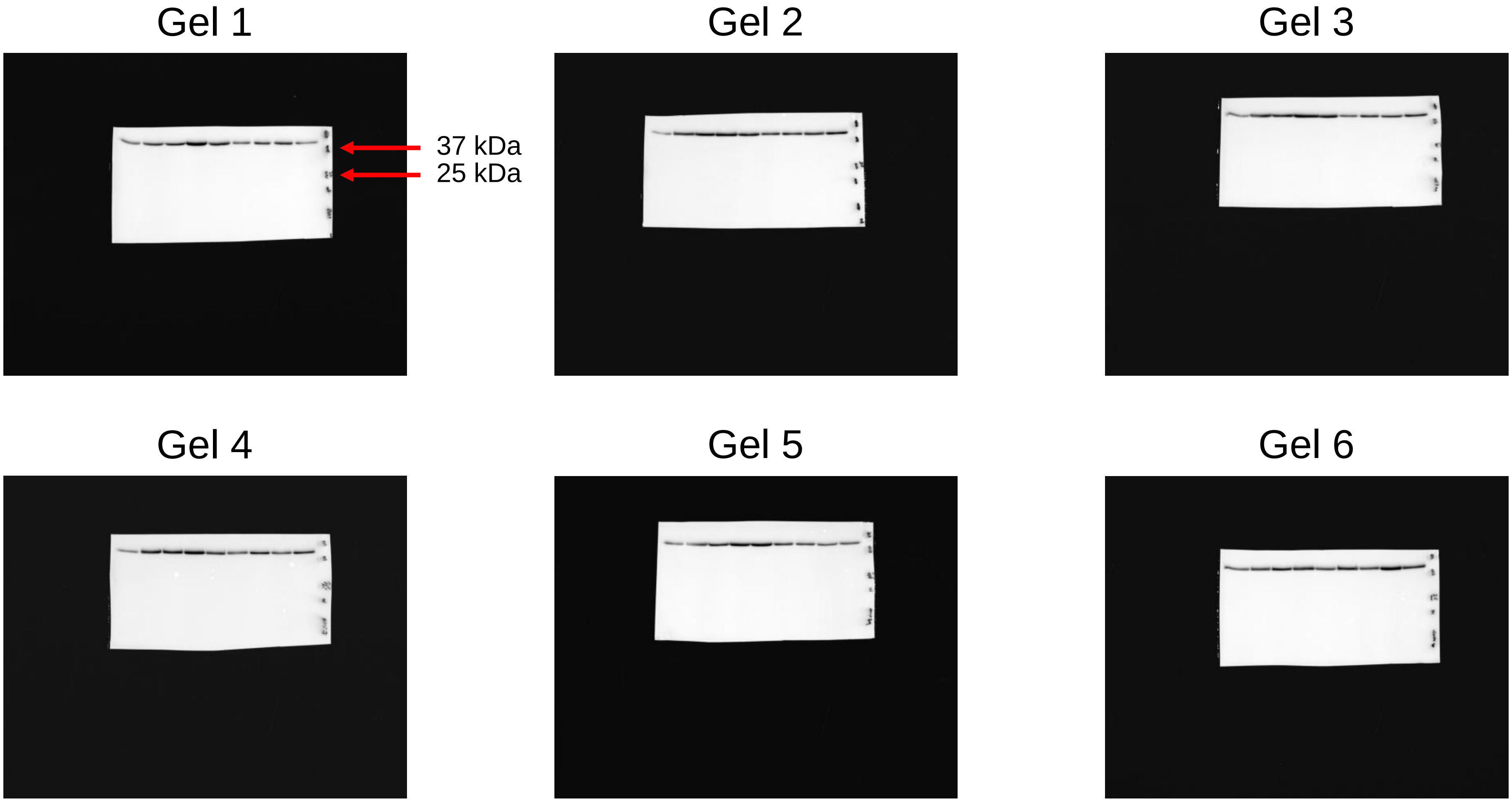

Gels: 12% acrylamide

A3854, Millipore-Sigma (1:5000)

How to read the uncropped/unrotated membranes: Set 2 loading

In set 1, Dahl S samples, for an unknown reason, saturated the images and decreased the pixel density of Dahl R samples. Therefore, in set 2, Dahl R samples were run on their on gels.

NF: Non-fasting
F: Fasting
LS: Low-salt
HS: High salt

F	NF	F	NF	F	NF	F	NF
HS		LS		HS		LS	
Dahl R sample 2				Dahl R sample 1			

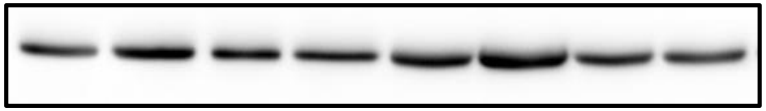

LADDER

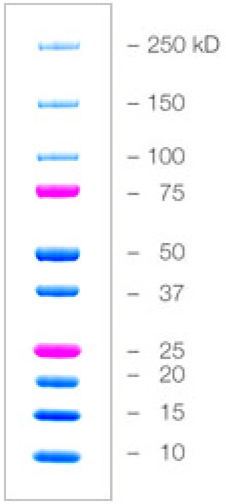

161-0374, Bio-rad (15 µl)

Hepatic LC3B-I and -II ~19 and 17 kDa

Gel 1

Dahl R

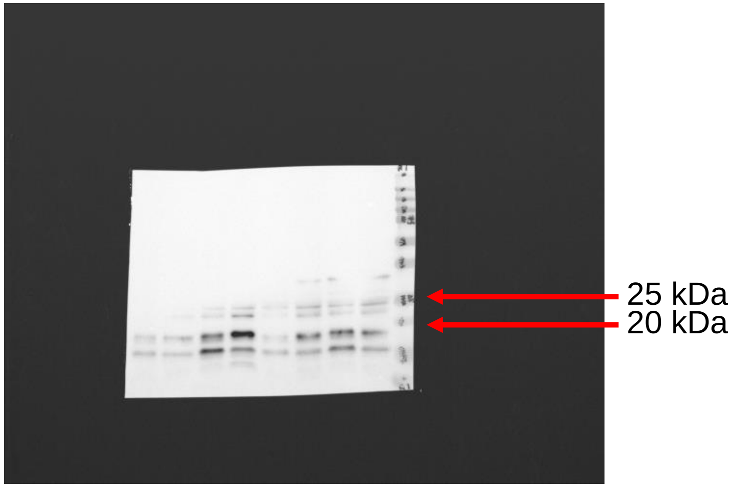

Gel 2

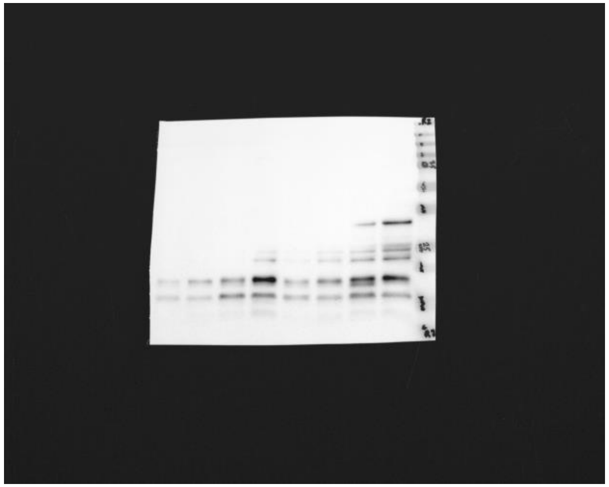

Gel 3

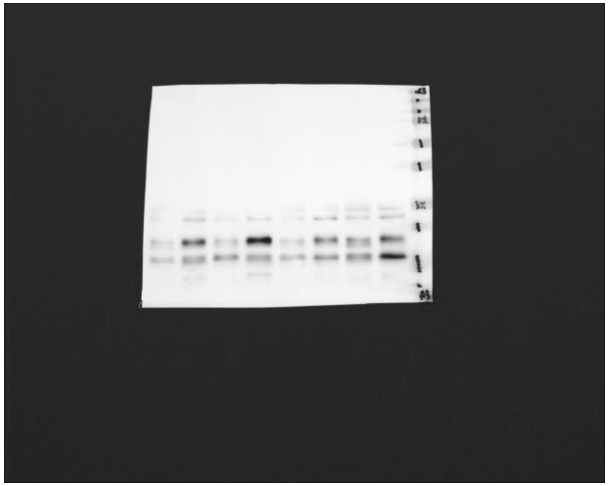

Gel 4

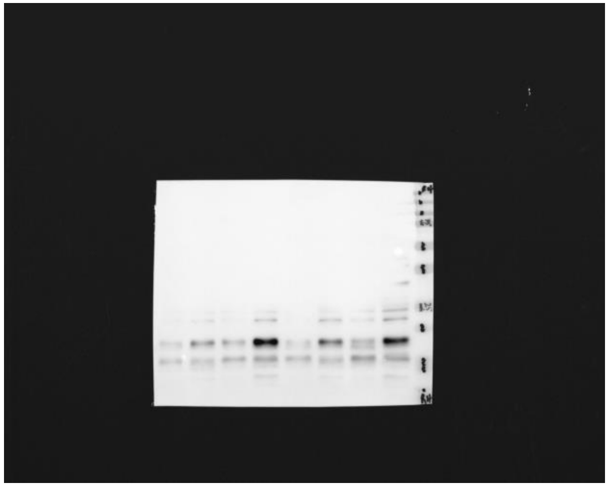

Gels: 12% acrylamide

NB600-1384, Novus Biologicals (1:1000)

Hepatic β actin– 42 kDa

Dahl R

Gel 1

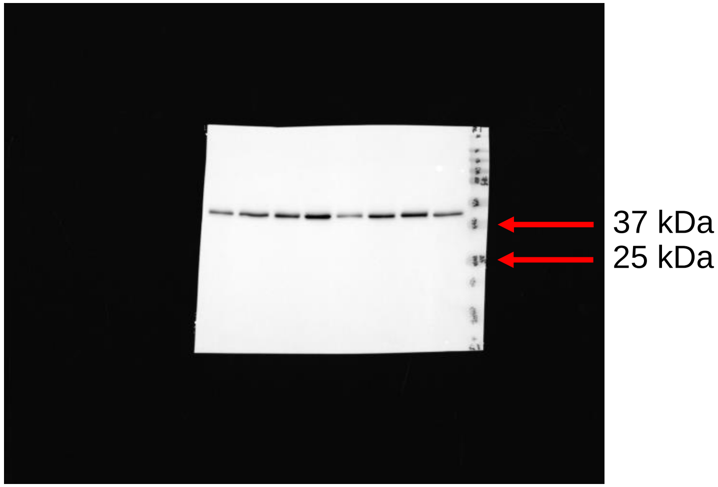

Gel 2

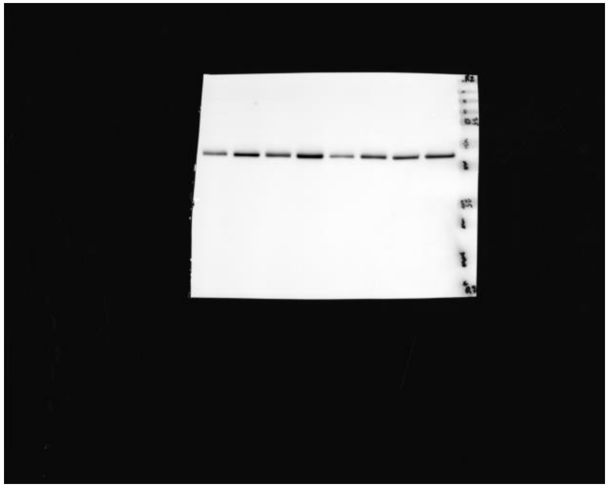

Gel 3

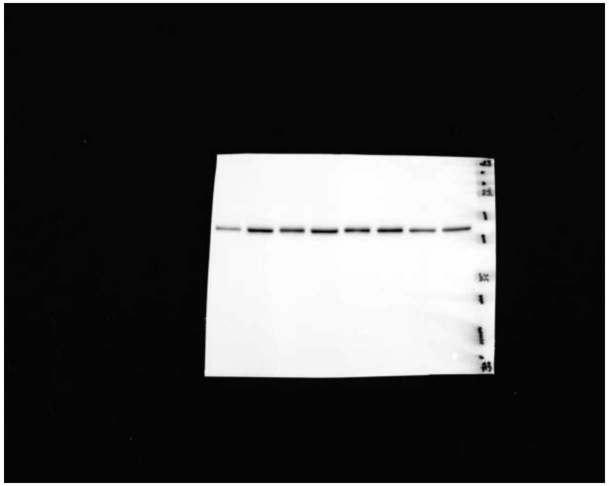

Gel 4

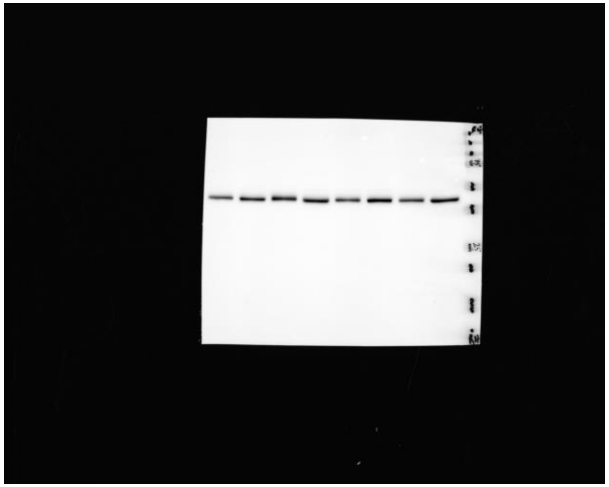

Gels: 12% acrylamide

A3854, Millipore Sigma (1:5000)

How to read the uncropped/unrotated membranes: Set 3 loading

Similar to set 1, Dahl S samples, for an unknown reason, saturated the images and decreased the pixel density of Dahl R samples. Therefore, Dahl R samples were excluded from analysis.

NF: Non-fasting
F: Fasting
LS: Low-salt
HS: High salt

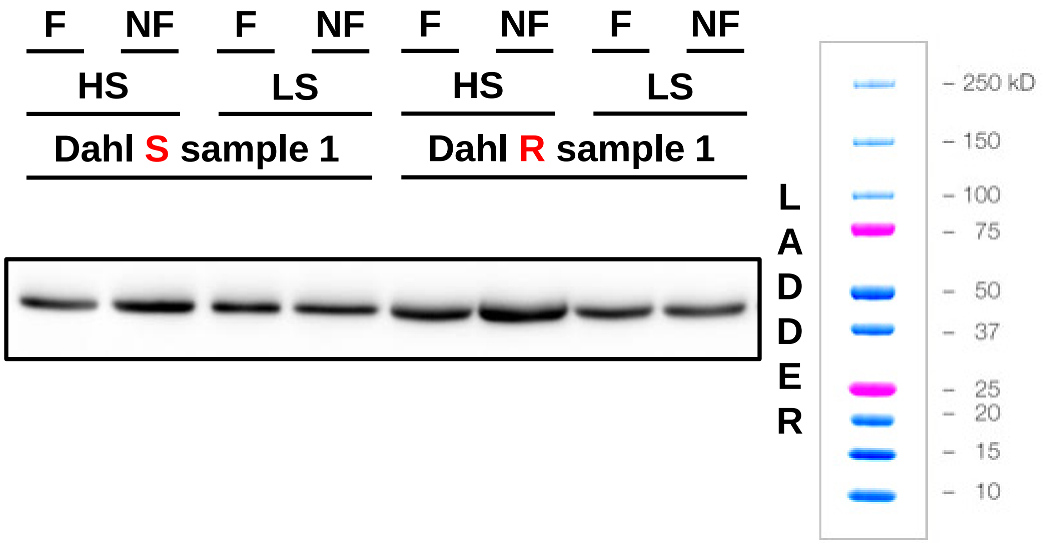

161-0374, Bio-rad (15 µl)

***Dahl S and Dahl R were analyzed independently**
****Gel 6 consisted of two sets of Dahl S samples, and no Dahl R**

p62 – 62 kDa

Gel 1

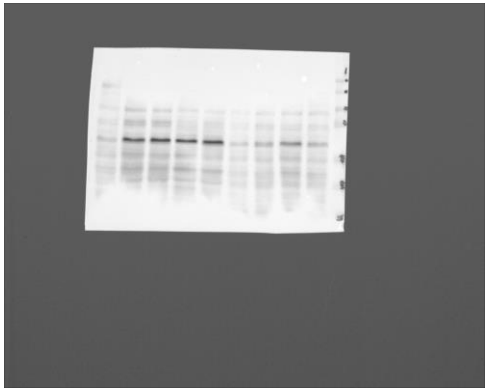

Gel 2

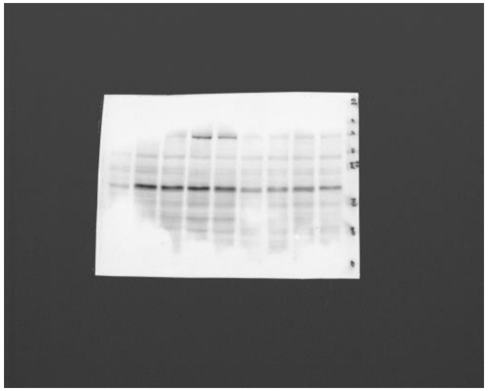

Gel 3

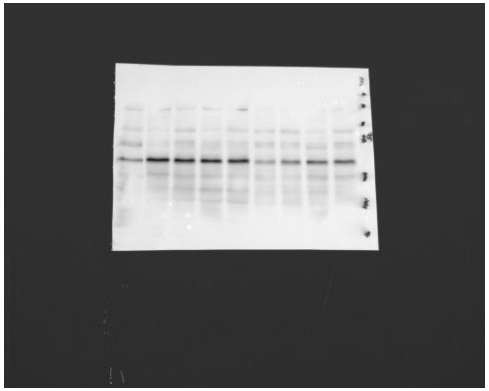

Gel 4

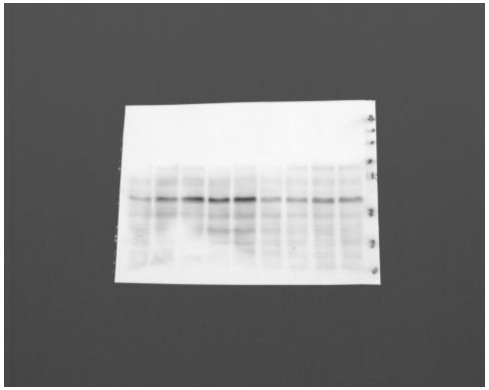

Gel 5

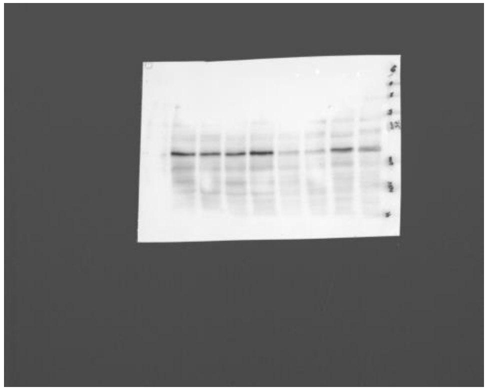

Gel 6

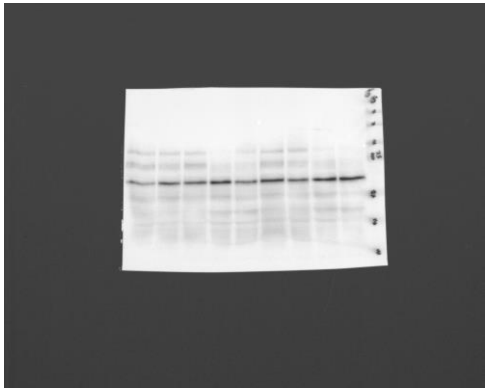

β actin – 42 kDa

Gel 1

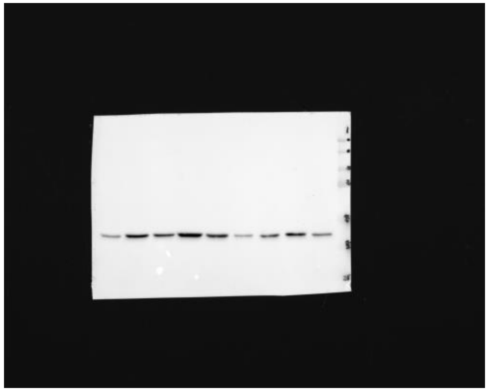

Gel 2

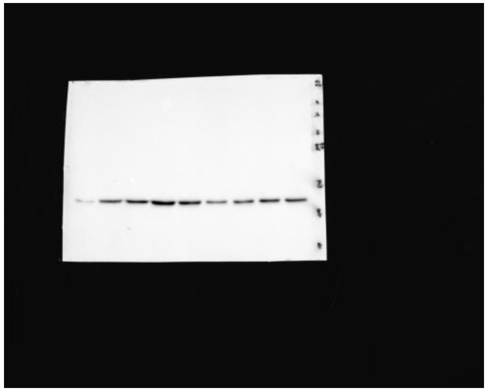

Gel 3

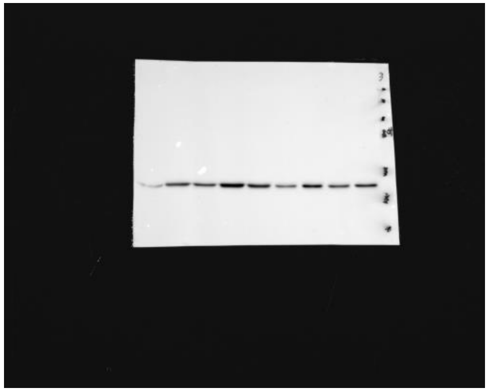

Gel 4

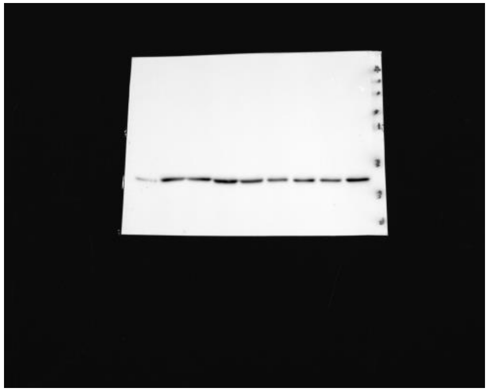

Gel 5

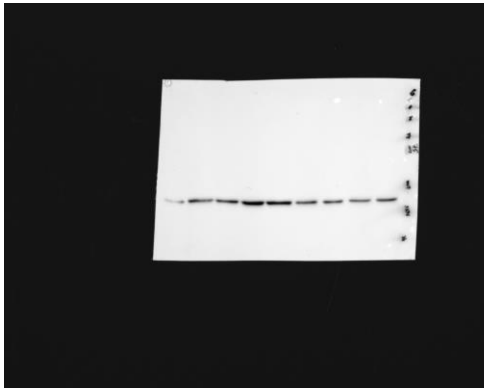

Gel 6

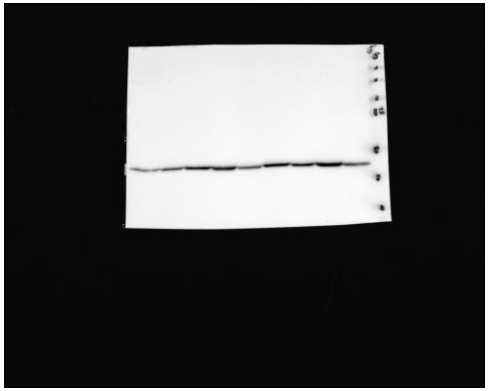

Supplement: Supplemental data [file jciinsight-6-149037-s047.pdf]
